# *Artemisia capillaris* with two novel active compounds, Kawarayomogin I and II, inhibits HYBID (KIAA1199) expression as well as hyaluronic acid degradation

**DOI:** 10.1038/s41598-025-86320-4

**Published:** 2025-01-15

**Authors:** Kazal Boron Biswas, Yuka Kawai, Satoshi Nakagawa, Kyoko Kanai, Hiroyuki Kojima, Teruaki Masutani, Masayoshi Oyama, Arunasiri Iddamalgoda, Kotaro Sakamoto

**Affiliations:** 1 Department of Research and Development, Ichimaru Pharcos Co. Ltd., Motosu, Gifu, Japan; 2 Laboratory of Pharmacognosy, Gifu Pharmaceutical University, Gifu, Japan

**Keywords:** *Artemisia capillaris* flower extract, HYBID, Hyaluronic acid, Kawarayomogin, miR-486-5p, Biochemistry, Health care

## Abstract

Hyaluronic acid (HA) is an important component of the skin’s extracellular matrix, and its degradation leads to wrinkles. Hyaluronan-binding protein involved in hyaluronan depolymerization (HYBID) is the main factor responsible for HA degradation in dermis. This study aimed to identify natural plant materials that can effectively suppress HYBID expression and protect HA from degradation. Screening of various plant extracts was performed for the inhibition of histamine-induced mRNA expression of HYBID in normal human dermal fibroblasts (NHDF). The molecular size distribution of HA was evaluated by incubating fluorescein isothiocyanate (FITC)-labeled large HA (1200–1600 kDa) in NHDF for certain time followed by measuring different sizes of FITC-labeled HA in the cultured medium by HPLC. Among 380 plant extracts, we found that *Artemisia capillaris* flower extract (ACFE) was the most effective agent in both suppressing HYBID expression as well as protecting large HA from degradation. Subsequent mechanism elucidation studies showed that ACFE epigenetically regulates the expression of HYBID by modulating the expression of a specific miRNA, miR-486-5p, which is known to directly target and inhibit HYBID expression. Our active compound search identified 1-caffeoyl-3-hydroxybutane and 3-caffeoyl-1-hydroxybutane in ACFE as new compounds, which we named Kawarayomogin I and Kawarayomogin II, respectively. This is the first report to show that *Artemisia capillaris* with two novel active compounds inhibits HYBID expression as well as hyaluronic acid degradation, and therefore, could be used as possible agent for cosmeceutical potential.

**Supplementary Information:**

The online version contains supplementary material available at 10.1038/s41598-025-86320-4.

## Introduction

Hyaluronic acid (HA), a naturally occurring non-sulfated glycosaminoglycan, is involved in many cellular and physiological processes (e.g., proliferation, differentiation, development, migration, lubrication, hydration balance, space-filling, and matrix structure)^1,2^. It is distributed ubiquitously through the extracellular matrices of various tissues, including skin, cartilage, and the brain^3,4^. Approximately half of the total HA in the body is in the dermis of the skin^5^, where it is synthesized by dermal fibroblasts^6^.

The balance between the synthesis and degradation of HA is vital to skin homeostasis, which is lost during aging. To maintain this balance, the catabolism of HA plays a major role compared to its counterpart anabolism because aging causes increased expression of degrading machineries and decreased expression of those responsible for synthesis. Hyaluronidase enzymes (HYAL) are known to be involved in the degradation of HA but their contribution to HA metabolism is unclear^7^. Recently, a new mechanism of HA degradation has been proposed, which is independent of HYAL. This novel mechanism involves a protein called HYBID (hyaluronan-binding protein involved in hyaluronan depolymerization) (also known as KIAA1199 and CEMIP1 [cell migration inducing protein 1]), which binds to and degrades HA with high-molecular weight (> 1000 kDa) into ones with low-molecular weight ranging from 10 to 100 kDa^7^. Recent studies have demonstrated that HYBID, but not HYAL, is the main player involved in the degradation of HA in human skin fibroblasts^7–9^. It has also been found that HYBID expressions are negatively correlated with HA levels and positively correlated with sagging and wrinkles in photo-aged skin, suggesting that HYBID-mediated HA degradation in dermis contributes to the development of wrinkles and sagging in such skin^10^.

HYBID is expressed in many cell types, including astrocytes^11^, fibroblasts^7^, synoviocytes^3^, osteoblasts^12^, and various cancer cells^13,14^. It is involved in the pathogenesis of various diseases, including rheumatoid arthritis and angiogenesis. The expression of HYBID in skin fibroblasts is regulated by factors such as cytokines and chemokines^7^ as well as UV^15^. Its expression is significantly upregulated by treatment with histamine^7^ which is usually released from mast cells upon ultraviolet (UV) irradiation. Recently, it has been reported that the expression of HYBID is increased in photo-aged skin, and this increase is probably due to the increased release of histamine from mast cells^10^. However, although the regulation of HYBID expression in extrinsic skin aging is well-documented^10^, there has been no research to date on its role in natural or intrinsic aging of the skin.

Apart from the histamine-mediated molecular mechanism of HYBID upregulation (through the histamine-HRH1-PKC pathway), microRNA (miRNA)-based regulation of HYBID expression has recently been demonstrated in some in vitro studies^16–20^. By definition, miRNAs are small, single-stranded, non-coding RNA molecules containing 19–25 nucleotides that are thought to regulate gene expression post-transcriptionally by binding to the 3’ untranslated region (UTR) of target mRNAs and inhibiting their translation^21^. Recent experimental evidence suggests that there are more than 800 unique miRNAs in humans^22^. However, sporadic studies indicate that a specific miRNA, miR-486-5p, directly targets HYBID mRNA and inhibits its translation to protein^16–20^. Although miR-486-5p has been reported to be involved in the pathogenesis of cancer in various organs, including the liver, lung, and breasts^16–20^, its expression and role in skin cells has not been investigated, particularly in human dermal fibroblasts. It is also important to elucidate its regulation of expression by various stimuli such as histamine.

Plants are a rich source of biologically active compounds, and plant-derived products are widely used all over the world as herbal medicines, topical applications, and health foods. The purpose of the present study was to find natural ingredients along with their active components that can inhibit HA degradation via direct suppression of HYBID expression and/ or via indirect increase of the HYBID inhibitor, miR-486-5p.

## Materials and methods

### Cell cultures

Normal human dermal fibroblasts (NHDF) from newborns (NB) and adults (AD) were purchased from Kurabo Industries Ltd. (Osaka, Japan). They were cultured in a 75 cm^2^ flask using Dulbecco’s Modified Eagle Medium (DMEM) (Fujifilm Wako Pure Chemical Corporation, Tokyo, Japan) and supplemented with 10% (vol/vol) fetal bovine serum (FBS) (Sigma–Aldrich Corporation, MO, USA) and a solution of 100 units/mL penicillin, 100 µg/mL streptomycin, and 0.25 µg/mL amphotericin B (Antibiotic-Antimycotic, Gibco Life Technologies Corp., NY, USA). They were then maintained at 37 °C in an atmosphere of 95% air and 5% CO_2_. Unless otherwise mentioned, the cells in all experiments were seeded on 6-well plates (6 × 10^4^ cells/well) and cultured in DMEM containing 10% FBS. After the cells reached about 75% confluence, the medium in each well was exchanged for fresh medium containing 0.25% FBS and incubated for 24 h. Then, the cells were exposed with various concentrations of ACFE for 3 h and incubated with histamine (10 µM) for 24 h for gene expression analysis.

### Plant information

We screened 380 plant extracts^23,24^ to examine their ability to inhibit HYBID expression in newborn fibroblasts in the presence of histamine. For the screening, the cells were incubated with various extracts for 3 h, and then challenged with histamine (10 µM) for further 24 h before isolating RNA and measuring HYBID expression. After the screening, we primarily selected 3 extracts based on their highest efficacies to inhibit HYBID. Then, we investigated the concentration-dependent efficacies of those extracts and identified the plant, *Artemisia capillaris* Thunb. (Compositae), as a potent suppressor of HYBID with excellent consistency. It is known as a medicinal plant throughout the world, and we procured the flowering parts of the plant from Tochimoto Tenkaido, Osaka, Japan. The lot number of the material is 020820002, and it is stored under a constant temperature of 25^0^C. The flowers of the plant were extracted in 30% butylene glycol (BG) and purified using a 0.45 μm cellulose acetate membrane filter (Advantec Toyo Roshi Kaisha, Ltd., Tokyo, Japan). The extract was named *Artemisia capillaris* flower extract and denoted as ACFE throughout the whole manuscript.

### Identification of the active compounds in ACFE

For the fractionation of ACFE, water was added to obtain an aqueous ACFE solution containing 15% BG. The solution was then passed through an open column filled with a nonpolar copolymer styrene-divinylbenzene adsorbent resin, DIAION HP-20 (Mitsubishi Chemical Corporation, Japan), and four fractions were obtained: 15% ethanol-eluted fraction (Fr.1), 30% ethanol-eluted fraction (Fr.2), 45% ethanol-eluted fraction (Fr.3), and 100% ethanol-eluted fraction (Fr.4). Fr.2 was then further fractionated using preparative HPLC to obtain four sub-fractions (Fr.2.1, Fr.2.2, Fr.2.3, and Fr.2.4). Two active fractions, Fr.2.2 and Fr.2.3 (each consisting of a single peak) were identified as compound 1 and compound 2, respectively. The HPLC condition and other spectroscopic details have been demonstrated in the Supplementary Materials.

### Total RNA extraction

For mRNA determination, total RNA was extracted from newborn and adult fibroblasts using an Rneasy Mini Kit (Qiagen, CA, USA) according to the manufacturer’s instructions. For miRNA analysis, miRNeasy Tissue/ Cells Advanced Mini Kit (Qiagen, CA, USA) was used to isolate total RNA, including miRNA, from fibroblasts according to the manufacturer’s instructions.

### Real-time polymerase chain reaction (PCR)

For the measurement of mRNA, we followed the protocol as described in our previous study^25^. The primer set ID for HYBID is HA243626 (Takara Bio, Otsu, Japan).

For the measurement of miRNA, total RNA was reverse transcribed to complementary DNA (cDNA) using a Mir-X miRNA First-Strand Synthesis Kit (Takara Bio, Otsu, Japan). Relative semi-quantitative real-time PCR was carried out using the Mir-X miRNA qRT-PCR SYBR Kit (Takara Bio, Otsu, Japan) according to the manufacturer’s instructions. The sequence for miR-486-5p specific primer was 5´- TCCTGTACTGAGCTGCCCCGAG-3´. This was used as a forward primer. The universal primer, mRQ 3´, supplied with the PCR kit, served as a reverse primer. Delta-delta-CT method was used to compare the expressions in different groups.

### Measurement of HA size using the chromatographic method

After the addition of ACFE to cells, fluorescein isothiocyanate (FITC)-labeled large HA (FA-HA-H1; 1,200-1,600 kDa) (Iwai Chemicals Co., Ltd., Tokyo, Japan) was treated in the presence or absence of histamine. After 24 h of incubation, the cultured medium was collected for HA measurement by HPLC using a TSKGL G5000 PW-XL column (Tosoh, Tokyo, Japan) equilibrated with 0.2 mol/L NaCl in distilled water. The flow rate was 0.5 mL/min, and FA-HA was detected at an excitation wavelength of 490 nm using an emission wavelength of 525 nm. The degradation of large HA was analyzed by measuring the height (size) of the chromatographic peak of the intact and degraded HA present in the cultured medium.

### Statistical analysis

Data were expressed as mean ± standard deviation (M ± SD), and analyzed by JMP8 (SAS Institute, NC, USA) statistical software. Statistical analyses used Dunnett’s test, and Student’s t-test, as appropriate. A *p*-value of < 0.05 was considered statistically significant.

## Results and discussion

**Fig. 1:**
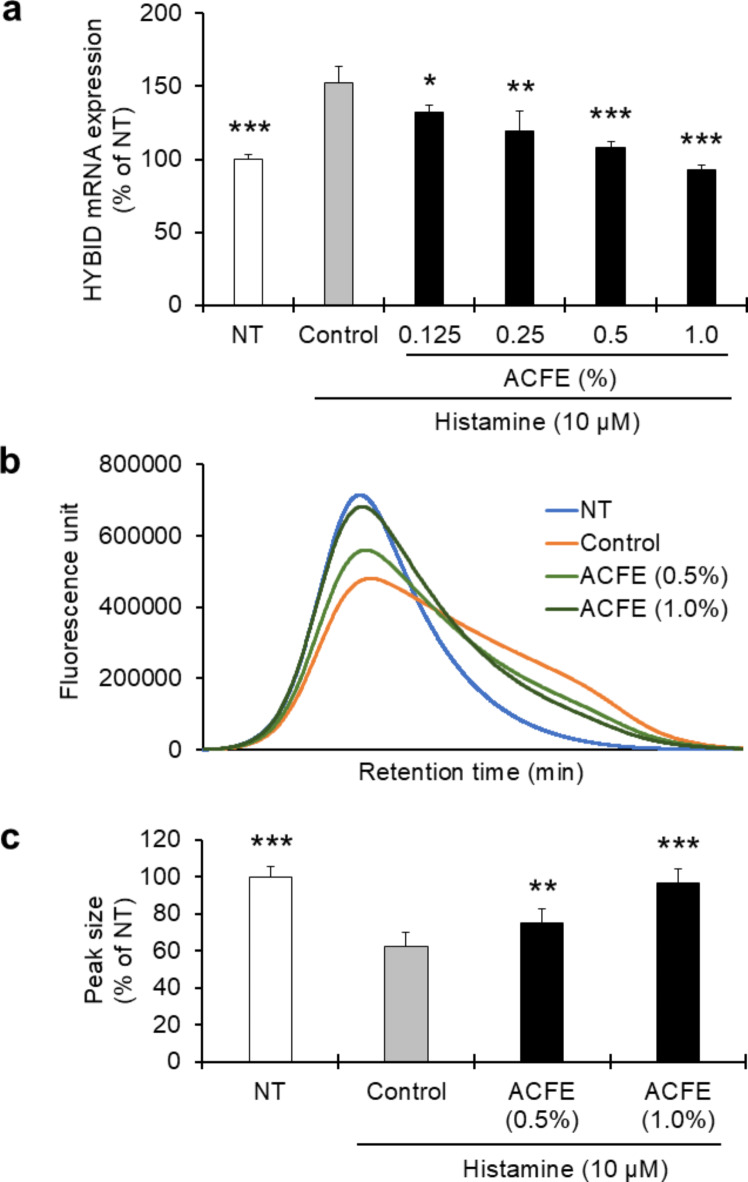
Effects of ACFE on the suppressions of HYBID as well as HA degradation in fibroblasts. (**a**) Different concentrations (0.125, 0.25, 0.5, and 1.0%) of ACFE were treated with newborn fibroblasts for 3 h and then challenged with histamine (10 µM) for 24 h followed by HYBID measurement by PCR. Data were expressed as mean ± SD (*n* = 3) and analyzed by Dunnett’s test (*p < 0.05, **p < 0.01, and ***p < 0.001 vs. control). (**b**) Both ACFE-treated and -untreated fibroblasts were cultured in the presence or absence of histamine as well as FITC-tagged large HA (MW, 1,200–1,600 kDa). After 24 h, the cultured medium was collected, and HA was measured by HPLC. The representative figure shows the size distribution of HA. Higher peaks indicate large HA while lower peaks denote degraded HA; (**c**) The height of the peaks in the NT (no treatment) group was considered to be 100% and the heights of the peaks in the other groups were calculated through comparison with this NT value. Data were expressed as mean ± SD (*n* = 8) and analyzed by Dunnett’s test (**p < 0.01 and ***p < 0.001 vs. control). ACFE, *Artemisia capillaris* flower extract; NT, No Treatment.

Different concentrations (0.125, 0.25, 0.5, and 1.0%) of ACFE were treated with newborn fibroblasts for 3 h, then removed, and treated with histamine (10 µM). Following 24 h of histamine challenge, the MTT (3-[4,5-dimethylthiazol-2-yl]-2,5 diphenyl tetrazolium bromide) cell viability assay and real-time PCR were carried out. We found that the ACFE inhibits the histamine-induced mRNA expression of HYBID in newborn fibroblasts in a concentration-dependent manner (Fig. 1a). However, there is no cellular toxicity observed at any of the doses (Supplementary Fig. S1).

As HYBID is responsible for the degradation of large HA, we measured the HA size distribution in newborn fibroblasts in the absence or presence of ACFE. As expected, the addition of histamine increased the rate of large HA degradation, but the treatment with ACFE dose-dependently decreased the histamine-induced degradation of large HA (Fig. 1b and c). As histamine induces HYBID expression and ACFE suppresses histamine-induced HYBID, it is likely that the reduction of histamine-induced HA degradation by ACFE is mediated by a HYBID-regulated mechanism. We also found that the degradation of HA increases over time, with higher rates of degradation at 48 h than at 24 h, under both normal (non-stimulatory) and histamine-stimulation conditions. ACFE (0.5 and 1.0%) significantly protected large HA from degradation even after 48 h (Supplementary Fig. S2). Recent reports demonstrate that UV increases the expression of HYBID both at cellular^15^ and tissue level^10^. UV is commonly known to increase the release of histamine from mast cells which, in turn, binds to the histamine-receptor on the membrane of various skin cells, for example fibroblasts, and causes adverse effects on skin. In our study, histamine increases HYBID expression as well as HA breakdown in newborn fibroblasts, and ACFE dose-dependently inhibits the action of histamine. Therefore, based on our data and recent published reports on UV-histamine-HYBID-HA inter-relationship, we assume that ACFE could inhibit UV-induced HA degradation and might be effective against extrinsic aging.

**Fig. 2:**
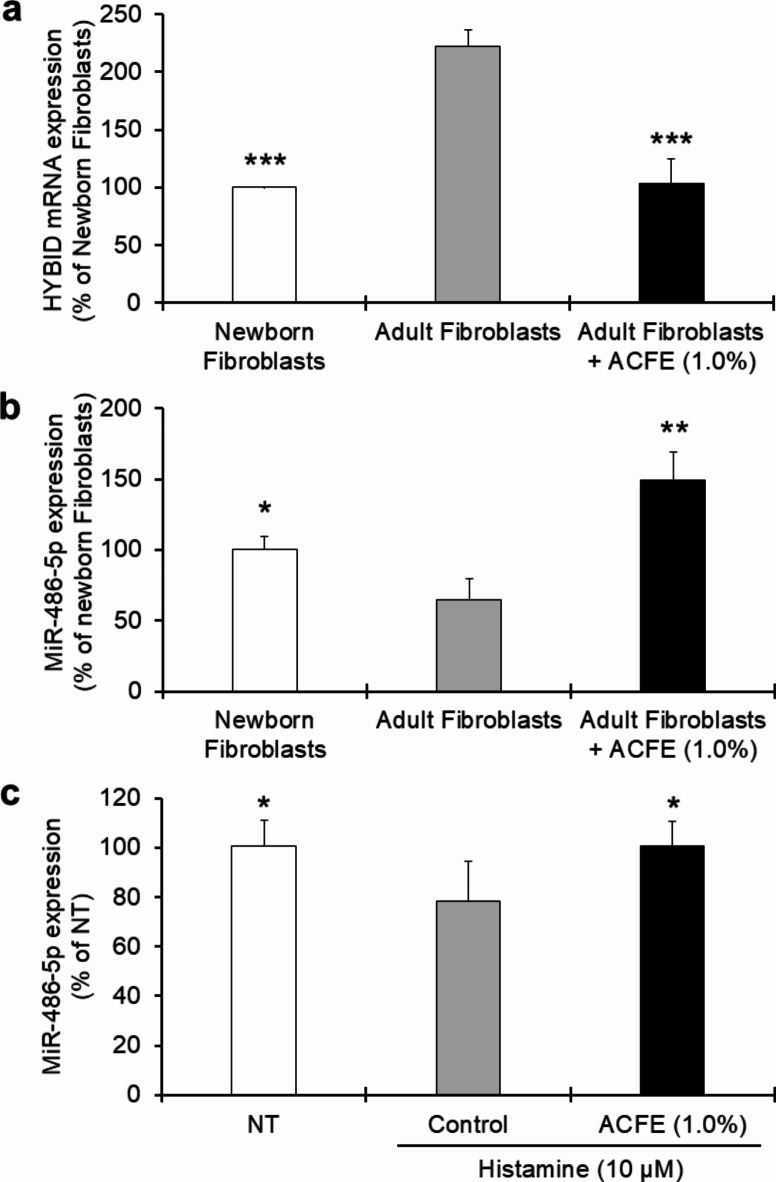
Effects of ACFE on the expressions of HYBID and its counterpart, miR-486-5p, in newborn and adult fibroblasts. Newborn and adult fibroblasts were exposed with ACFE for 3 h. Then, the medium was exchanged with a fresh one, and further incubated for 24 h for the measurement of HYBID (**a**) and only 6 h for the measurement of miR-486-5p (**b**) expressions. Data were expressed as mean ± SD (*n* = 3) and analyzed by Student’s t-test (*p < 0.05, **p < 0.01, and ***p < 0.001 vs. Adult Fibroblasts). (**c**) Newborn fibroblasts were cultured in the presence of ACFE for 3 h followed by addition of histamine for 6 h for the measurement of miR-486-5p. Data were expressed as mean ± SD (*n* = 6) and analyzed by Dunnett’s test (*p < 0.05 vs. Control). NT, No Treatment; ACFE, *Artemisia capillaris* flower extract.

Increased HYBID expression has been reported in photo-aged skin^10,26^, however, there is no research on its expression in naturally aged skin. Therefore, we investigated the relative expression of HYBID in adult (derived from a female of 47 years) and newborn fibroblasts. We found that adult fibroblasts express significantly higher levels of HYBID mRNA than newborn fibroblasts. It is generally accepted that inflammation is a key factor in the aging process, and there are reports that certain proinflammatory mediators are involved in the over-expression of HYBID in osteoarthritic chondrocytes^27,28^ and pancreatic cancer cells^29^, therefore, we cannot exclude the possibility that the expression of HYBID in skin fibroblasts is regulated by inflammation caused by senescence. Interestingly, ACFE significantly suppressed HYBID expression in adult fibroblasts (Fig. 2a), but the detailed mechanism of how ACFE affects HYBID expression is not clearly understood. Considering the fact that the expression of HYBID is upregulated through histamine receptor-Protein Kinase C (PKC) signaling pathway in photo-aged skin^9^ and PKC activity increases with natural aging^30^, we may speculate that ACFE could block any cell surface receptors (PKC pathway may involve various cell surface receptors such as insulin receptor, IGF-1 receptor etc.) and, thereby, suppress the PKC downstream pathways responsible for HYBID expression in both intrinsic and extrinsic aging of cells. Moreover, we investigated the expression of a microRNA, miR-486-5p, in skin cells and found that this miRNA is expressed in fibroblasts under normal physiological conditions. MiR-486-5p acts as a direct target of HYBID to inhibit its expression which is reported in several types of cancer cells^17–20^. In our study, adult fibroblasts express significantly lower levels of this miRNA than newborn fibroblasts. However, ACFE was found to increase the expression of this miR-486-5p in adult fibroblasts under normal cellular conditions (Fig. 2b). It is difficult to understand why miR-486-5p decreases with aging. While increased DNA methylation is reported to down-regulate the expression of this miRNA in different cell types^31,32^, there is no report on the involvement of any inflammatory pathways in the down-regulation of miR-486-5p. Consequently, it appears difficult to draw any concrete conclusion on the ACFE’s mode of action on the increase of miR-486-5p, and further studies are needed to achieve the goal. Furthermore, whether histamine has any effect, we checked the expression of this miRNA in the presence of histamine in newborn fibroblasts. We found that histamine decreases significantly the expression of miR-486-5p, and ACFE is able to increase the histamine-mediated down-regulation of this miRNA (Fig. 2c). However, it is not conceivable whether the expressions of miR-486-5p and HYBID are regulated by two independent mechanisms, or they follow the same regulatory pathway.

**Fig. 3:**
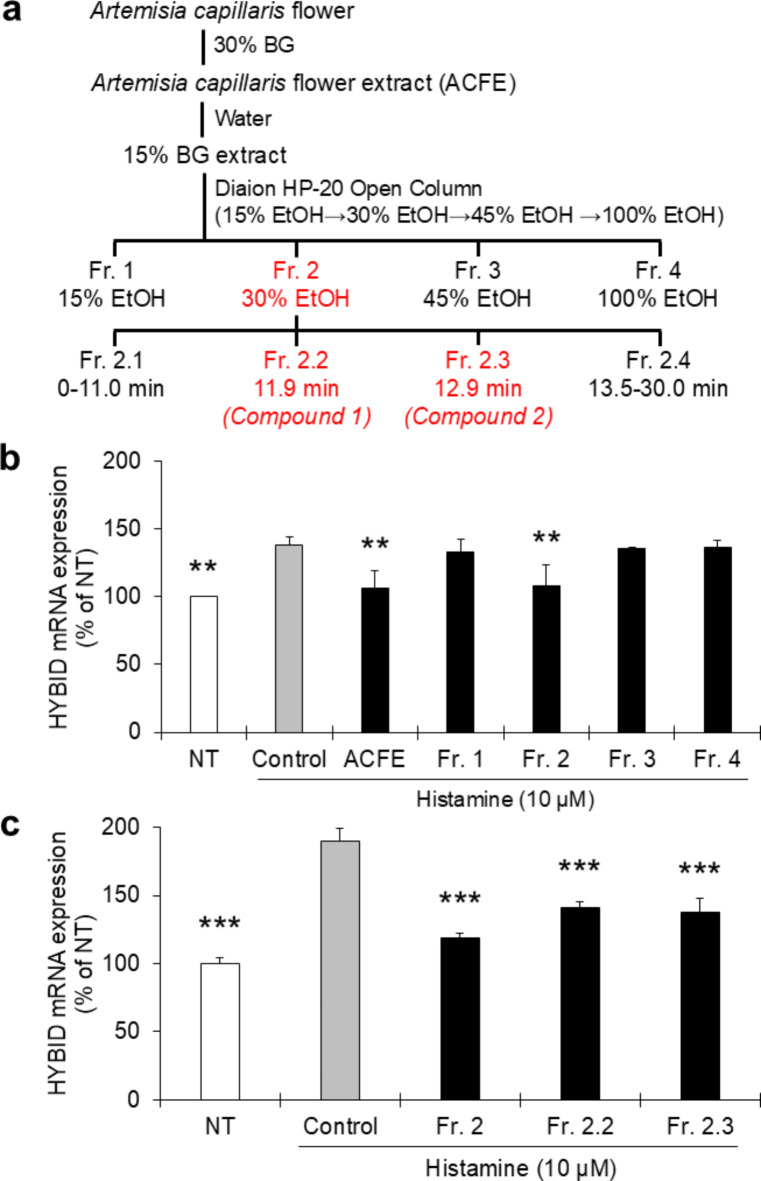
Identification of the active compounds in ACFE. (**a**) Flowchart showing the process by which various fractions of ACFE were isolated and purified; (**b**) The effects of four different fractions (Fr. 1 to Fr. 4) of ACFE on the inhibition of histamine-induced HYBID expression in newborn fibroblasts. Final concentrations of each fraction of 0.5% were evaluated for their efficacy in HYBID inhibition; (**c**) Among the further isolated sub-fractions, the effects Fr. 2.2 and Fr. 2.3 on the inhibition of histamine-induced HYBID expression in newborn fibroblasts were shown; To ensure equal concentrations of each fraction, the concentrations were adjusted through comparison with the same chromatographic peak area of active fraction in the main ACFE. HYBID expression in the NT (no treatment) group was taken to be 100% and its expression in other groups was calculated by comparison with this NT value. Data were expressed as mean ± SD and analyzed by Dunnett’s test. **p < 0.01, and ***p < 0.001 vs. control. ACFE, *Artemisia capillaris* flower extract; NT, No Treatment.

**Fig. 4:**
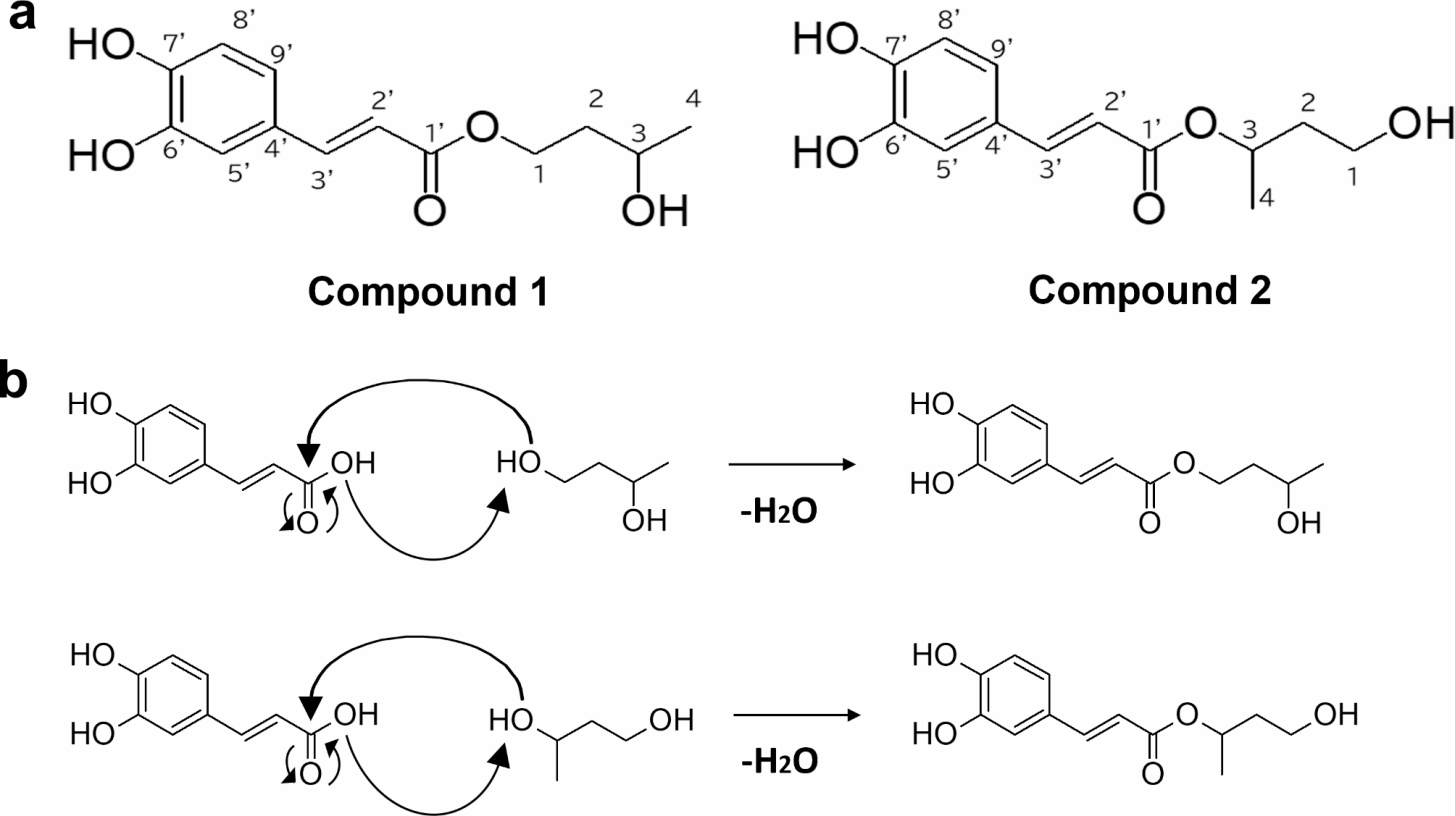
The structures of the two active components identified in ACFE. (**a**) Compound 1 was named Kawarayomogin I, and compound 2 was named Kawarayomogin II. The chemical names of these compounds are 1-caffeoyl-3-hydroxybutane and 3-caffeoyl-1-hydroxybutane, respectively. (**b**) Proposed mechanism of condensation of caffeic acid with butylene glycol.

To identify its active components, ACFE was purified and fractionated according to the flow chart shown in Fig. 3a. As 1% ACFE showed excellent efficacy in suppressing HYBID without showing any cellular toxicity, the same concentration was used to determine the efficacy of each fraction in the subsequent experiment. Depending on the ability of each fraction to suppress histamine-induced HYBID expression, we selected the active fraction containing a single chromatographic peak. Each purified fraction was prepared so that its corresponding peak area was comparable with the peak area of the original extract. Among the four fractions isolated, Fr. 2 showed almost total inhibition of HYBID expression (Fig. 3b). Therefore, Fr. 2 was further separated into four sub-fractions (Fr. 2.1 to Fr. 2.4). We found Fr. 2.2 and Fr. 2.3 to inhibit histamine-induced HYBID expression in newborn fibroblasts (Fig. 3c). Through NMR analysis of these two fractions, we identified two novel compounds the structures and names of which are shown in Fig. 4. The chemical names of the compounds are 1-caffeoyl-3-hydroxybutane and 3-caffeoyl-1-hydroxybutane, based on the Japanese name of the plant, we named these compounds Kawarayomogin I and Kawarayomogin II, respectively. As these are completely new compounds, they are not commercially available to be used as reference standards. Therefore, it was not possible to compare our data with reference compounds (positive control). The detailed results, including the spectral data were provided in the Supplementary Materials (Supplementary Figs. S3–S26).

The *Artemisia capillaris* is a well-known medicinal herb, with many reports of isolated active compounds from the extracts in the past such as p-hydroxyacetophenone, β-sitosterol, 4-methyl capillarisin, cirsilineol, quercetin, scoparone, cirsimaritin, arcapillin, capillin, capillone, capillarin, cirsimaritin, and capillarisin^33^. In this study, we reported two findings. First, we demonstrate that ACFE effectively suppresses the upregulation of HYBID expression due to external stimuli and aging. Second, we identify 1-caffeoyl-3-hydroxybutane (Kawarayomogin I) and 3-caffeoyl-1-hydroxybutane (Kawarayomogin II) as the major compounds responsible for this activity. Two plant materials, *Geranium thunbergii* and *Sanguisorba officinalis*, have recently been reported to possess HYBID-suppressing ability^34,35^, however, no active compounds have so far been discovered having such activity. In our investigation, we were able to identify the active compounds in ACFE for the first time in our search for a potent HYBID suppressor. Interestingly, to the best of our knowledge, the presence of these compounds in ACFE has not been previously reported, and their structures are novel. One significant reason for this discovery is that we prepared the ACFE using BG, as Kawarayomogin I and Kawarayomogin II are thought to be semi-synthetic compounds produced by the dehydration condensation of caffeic acid with BG (Fig. 4b). BG has two chiral centers, which are reflected in the structures of Kawarayomogin I and Kawarayomogin II. Thus, we could not determine the absolute configuration of these compounds. It remains unclear why Kawarayomogin I and Kawarayomogin II were produced in the ACFE prepared with BG. We confirmed that simply mixing BG and caffeic acid does not produce Kawarayomogin I and Kawarayomogin II (data not shown). We have also confirmed that the addition of caffeic acid to the ACFE extracted with BG does not increase the amounts of Kawarayomogin I and Kawarayomogin II (data not shown). Some substances with catalytic activity might be contained in *Artemisia capillaris*, and they are involved in the reaction pathway leading to the production of Kawarayomogin I and Kawarayomogin II. However, the molecular mechanism remains unclear at this time and is a subject for future research.

Our data that ACFE suppresses both histamine-stimulated and histamine-non-stimulated expressions of HYBID in fibroblasts imply that a histamine-independent mechanism of HYBID expression may exist. We found that a specific miRNA, miR-486-5p, is upregulated by ACFE under non-stimulatory conditions. In fact, miR-486-5p is reported to be a directly targeted miRNA for the inhibition of HYBID expression^17,18^, and overexpression of this miRNA has been linked with a decreased risk of cancer in some tissues as well as accelerated skin wound healing^18,36^. Therefore, it is reasonable to think that the HYBID-suppressing effect of ACFE might be meditated through increasing the level of miR-486-5p expression under the same condition. On the other hand, our data that histamine inhibits the expression of miR-486-5p in newborn fibroblasts imply that this chemical agent may induce HYBID overexpression through inhibiting the expression of this miRNA at least in this cell type. Moreover, the lower expression of this miRNA in adult than in newborn fibroblasts correlates well with the higher expression of HYBID in adult than newborn fibroblasts found in our study (Fig. 2). Therefore, we may collectively speculate that ACFE, irrespective of histamine stimulation, might be able to upregulate the expression of miR-486-5p which in turn inhibits the expression of HYBID.

So far, the inhibition of HA degradation in various tissues including skin epidermal layer has been targeted by inhibiting hyaluronidase enzymes. But recent studies demonstrate that in some tissues like skin fibroblasts, HYBID is the main to degrade HA^7–9^, and there are almost no inhibitors of HYBID currently available. We are probably the first to discover two novel plant-based active compounds as HYBID inhibitors which may suppress the HA degradation in skin. However, the investigation on the inhibitions of HYBID over-expression and HA degradation by ACFE or its active components has not been performed directly in human skin. Therefore, future studies are needed to address this issue. Moreover, these discoveries would further facilitate future basic research involving HA and HYBID in other tissues as well. Finally, ACFE and its components could potentially be beneficial to the skin, and thus, this study might provide a safe and natural strategy for the development of new topical aging care agents.

## Electronic supplementary material

Below is the link to the electronic supplementary material.

Supplementary Material 1

## Data Availability

Data is provided within the manuscript or supplementary information files. Raw data are available from the corresponding authors upon reasonable request.
