# Supplementary material for: Artemisia capillaris with two novel active compounds, Kawarayomogin I and II, inhibits HYBID (KIAA1199) expression as well as hyaluronic acid degradation
Source: Sci Rep. 2025 Jan 15;15:2042. doi: 10.1038/s41598-025-86320-4 (PMC11739587; doi:10.1038/s41598-025-86320-4)
Supplement: Supplementary file 1 — Supplementary Material 1 [file 41598_2025_86320_MOESM1_ESM.pdf]

Supplementary Materials

Artemisia capillaris with two novel active compounds, Kawarayomogin I and II, inhibits HYBID (KIAA1199) expression as well as hyaluronic acid degradation

Kazal Boron Biswas[1*], Yuka Kawai[1], Satoshi Nakagawa[1], Kyoko Kanai[1], Hiroyuki Kojima[1], Teruaki Masutani[1], Masayoshi Oyama[2], Arunasiri Iddamalgoda[1], Kotaro Sakamoto[1*]

[1]Department of Research and Development, Ichimaru Pharcos Co. Ltd., Gifu, Japan;
[2]Laboratory of Pharmacognosy, Gifu Pharmaceutical University, Gifu, Japan

*Corresponding authors
Kazal Boron Biswas, Ph.D., Department of Research and Development, Ichimaru Pharcos Co. Ltd., Motosu, Gifu, Japan, Tel: 81-58-320-1017, Email: kazal@ichimaru.co.jp; ORCID ID: https://orcid.org/0000-0002-9678-5089

Kotaro Sakamoto, Ph.D., Department of Research and Development, Ichimaru Pharcos Co. Ltd., Motosu, Gifu, Japan, Tel: 81-58-320-1017, Email: sakamoto-kotaro@ichimaru.co.jp; ORCID ID: https://orcid.org/0000-0003-1295-1623

Supplementary method

Active components identification

<u>HPLC Conditions</u>

Instrument: LC-20ADxr-SPD-M20A (Shimadzu Corporation, Tokyo, Japan)

Column: Mightysil RP-18 GP 250 X 4.6 mm (5 μm) (Kanto Chemical Co. Inc., Tokyo, Japan)

Mobile phase: (acetonitrile: water) = (22: 78)/ 30 min

Flow rate: 1 mL/ min

Column temperature: 40°C

Detection: UV 280 nm

Compound 1 and compound 2 were subjected to NMR spectroscopy to determine their structures. For the structural analysis, ^{1}H–NMR, ^{13}C–NMR, COSY, HMQC, HMBC (JNM-ECA-500 spectrometer, JEOL Ltd., Tokyo, Japan), high-resolution electrospray ionization mass spectroscopy (HRESIMS) (LCMS-IT-TOF spectrometer, Shimadzu Corporation, Tokyo, Japan), optical rotations (P-1020 polarimeter, JASCO Corporation, Tokyo, Japan), and UV spectra (UV-3100 spectrometer, Shimadzu Corporation, Tokyo, Japan) were used. The spectra were as follows:

Compound 1: Yellow solid. [α]D + 4.0° (c = 0.1 g/dL, MeOH). UV λMeOH max nm

(logε): 218 (4.11), 243 (3.97), 329 (4.20). HRESIMS: calcd. For $C_{13}H_{15}O_5$ 251.0925 (M-H), found 251.0911. 1H NMR (500 MHz, MeOH-d4): δ = 7.53 (1 H, d, J = 15.8 Hz, 3'-H), 7.03 (1H, d, J = 1.9 Hz, 5'-H), 6.94 (1H, dd, J = 8.0, 1.9 Hz, 9'-H), 6.77 (1H, d, J = 8.0 Hz, 8'-H), 6.25 (1H, d, J = 15.8 Hz, 2'-H), 4.27 (2H, br t, J = 6.5 Hz, 1-H), 3.90 (1H, dqd, J = 8.0, 6.3, 4.6 Hz, 3-H), 1.83 (1H, dtd, 14.2, 7.1, 4.6 Hz, 2-Hb), 1.77 (1H, ddt, 14.2, 8.0, 6.0 Hz, 2-Ha), 1.21 (3H, d, J = 6.3 Hz, 4-H). 13C NMR (125 MHz, MeOH-d4): δ = 169.4 (C-1'), 149.6 (C-7'), 146.9 (C-6'), 146.9 (C-3'), 127.7 (C-4'), 122.9 (C-9'), 116.5 (C-8'), 115.10 (C-2'), 115.07 (C-5'), 65.5 (C-3), 62.7 (C-1), 39.0 (C-2), 23.8 (C-4).

Compound 2: Yellow oil. $[\alpha]_D$ + 3.6° (c = 0.1 g/dL, MeOH). UV λ_{max}^{MeOH} nm (logε): 217 (3.99), 234 (3.80), 243 (3.80), 300 (3.89), 328 (3.98). HRESIMS: calcd. For $C_{13}H_{15}O_5$ 251.0925 (M-H), found 251.0911. 1H NMR (500 MHz, MeOH-d4): δ = 7.52 (1H, d, J = 16.0 Hz, 3'-H), 7.03 (1H, d, J = 1.9 Hz, 5'-H), 6.94 (1H, dd, J = 8.2, 1.9 Hz, 9'-H), 6.77 (1H, d, J = 8.2 Hz, 8'-H), 6.24 (1H, d, J = 16.0 Hz, 2'-H), 5.12 (1H, dqd, J = 8.2, 6.3, 4.9 Hz, 3-H), 3.62 (2H, br t, J = 6.6 Hz, 1-H), 1.89 (1H, ddt, 14.0, 8.2, 6.6 Hz, 2-Hb), 1.81 (1H, dtd, 14.0, 6.6, 4.9 Hz, 2-Ha), 1.30 (3H, d, J = 6.3 Hz, 4-H). 13C NMR (125 MHz, MeOH-d4): δ = 169.0 (C-1'), 149.6 (C-7'), 146.8 (C-6'), 146.7 (C-3'), 127.7 (C-4'), 122.9 (C-9'), 116.5 (C-8'), 115.5(C-2'), 115.1 (C-5'), 69.5 (C-3),

59.3 (C-1), 39.9 (C-2), 20.6 (C-4).

Supplementary results

Active components identification

The molecular formulas of the compounds determined by HRESIMS were the same, $C_{13}H_{16}O_5$, but the configurations were different. The UV spectrum suggested a phenylpropanoid structure. In ^{1}H NMR, the peaks at 6.77, 6.94, and 7.03 ppm were found to have a trisubstituted benzene skeleton based on chemical shift, coupling, and J value. In addition, the peaks at 6.25 and 7.53 ppm were found to have a trans-olefin structure from the coupling and the J value at 15.8 Hz. Compound 1 appeared to have a phenylpropanoid skeleton. It was then estimated to have a dihydroxybutane skeleton based on the chemical shifts, coupling, J values, and ^{1}H integral values of peaks at 1.21, 1.77, 1.83, 3.90, and 4.27 ppm in ^{1}H NMR. In ^{1}H NMR, a peak at 4.27 ppm was found to be a methylene proton adjacent to the carboxyl group of phenylpropanoid from HMBC. From the above, we were able to infer that compound 1 is an ester bond of dihydroxybutane to phenylpropanoid, with the chemical name 1-caffeoyl-3-hydroxybutane. As we found no description of this compound in any relevant literature, it was determined to be newly discovered and was named Kawarayomogin I (Fig. 3).

The molecular formula of compound 2 determined by HRESIMS was also $C_{13}H_{16}O_5$, and the UV spectrum suggested a phenylpropanoid structure. In ^1H NMR, the peaks at 6.77, 6.94, and 7.03 were the same as those in compound 1, so compound 2 was thought to also have a phenylpropanoid skeleton. It was then estimated to have a dihydroxybutane skeleton based on the chemical shift, coupling, J value, and ^1H integral value of peaks at 1.30, 1.81, 1.89, 3.62, and 5.12 ppm in ^1H NMR. Subsequently, in ^1H NMR, the peak at 5.12 ppm was found to be a methine proton adjacent to the carboxyl group of phenylpropanoid because it had an HMBC correlation with the carbon of the carboxy group.

Since the methyl proton showing 1.30 ppm in ^1H NMR had an HMBC correlation with the carbon of methine, compound 2 was found to be 3-caffeoyl-1-hydroxybutane, which is an ester bond of dihydroxybutane to phenylpropanoid. This compound has also not been previously described and was a new compound, so was named Kawarayomogin II (Fig. 3).

Figure S1.

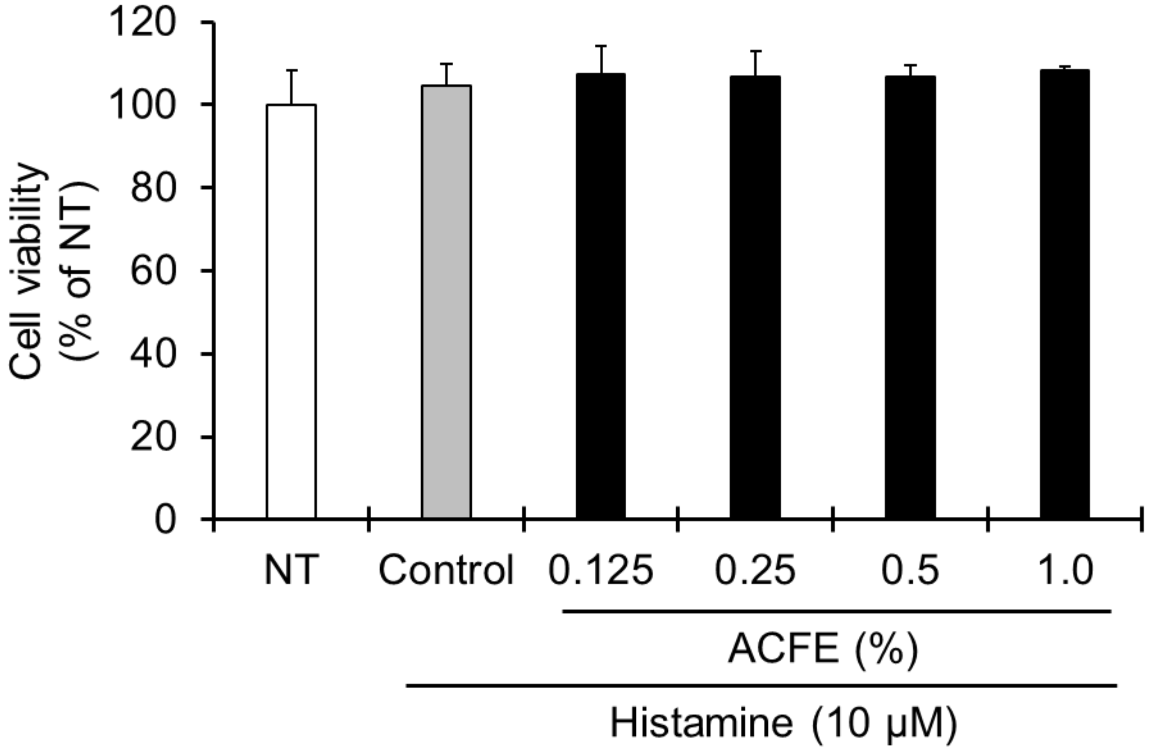

Figure S1. ACFE does not have any effect on the viability of newborn fibroblasts. Viability was measured by MTT method. Data were expressed as mean±SD (n=3) and analyzed by Dunnett's test.

Figure S2.

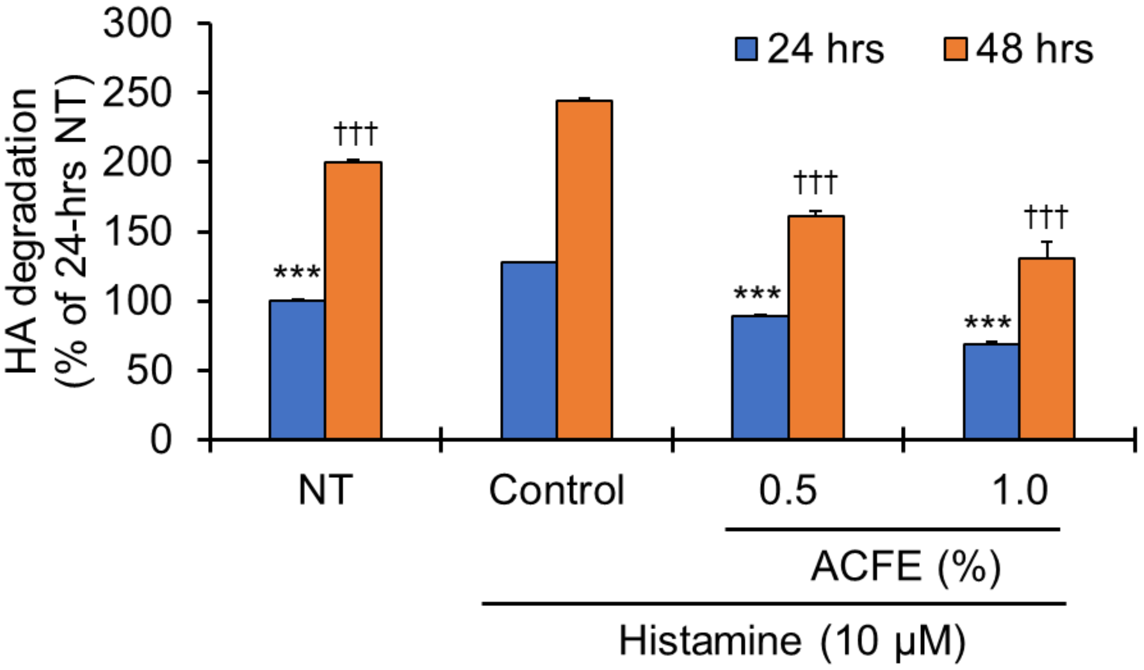

Figure S2. ACFE protects HA from degradation in newborn fibroblasts. ACFE treated- and untreated- fibroblasts were cultured in the presence or absence of histamine as well as FITC-tagged HA (M.W.: 1,200 – 1,600 kDa). After 24 and 48 hrs of incubation, cultured medium was collected for the measurement of HA by HPLC. Degradation of large HA was analysed by measuring the area under the curve of the intact and degraded HA. The area under the curve of HA-only (Untreated) for 24 hours was considered 100% and was then compared with others. It was found that histamine increased the HA degradation (increased small HA amount), but ACFE (0.5 and 1.0%) protected significantly the degradation of large HA (decreased small HA production) both at 24 and 48 hrs. Data were expressed as mean±SD (n=3) and analyzed by Dunnett's test (***p<0.001 vs Control-24 hrs; and †††p<0.001 vs Control-48 hrs).

Figure S3.

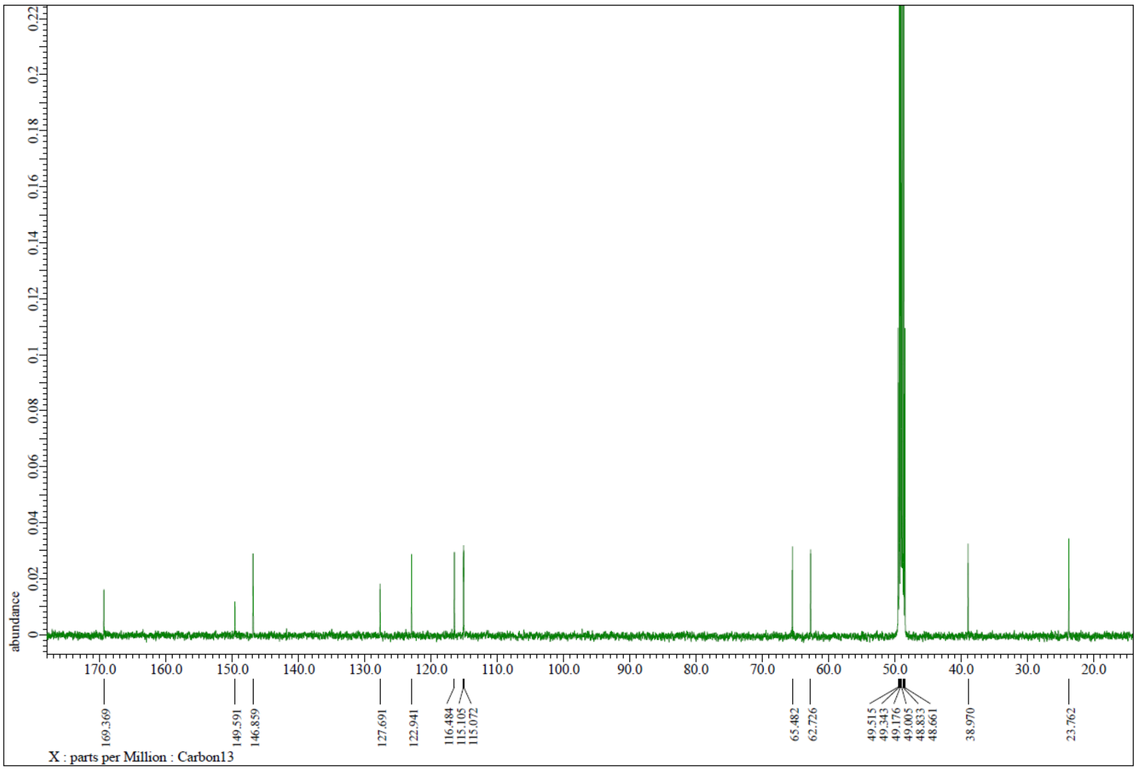

Figure S3. 13C NMR spectrum of Kawarayomogin I

Figure S4.

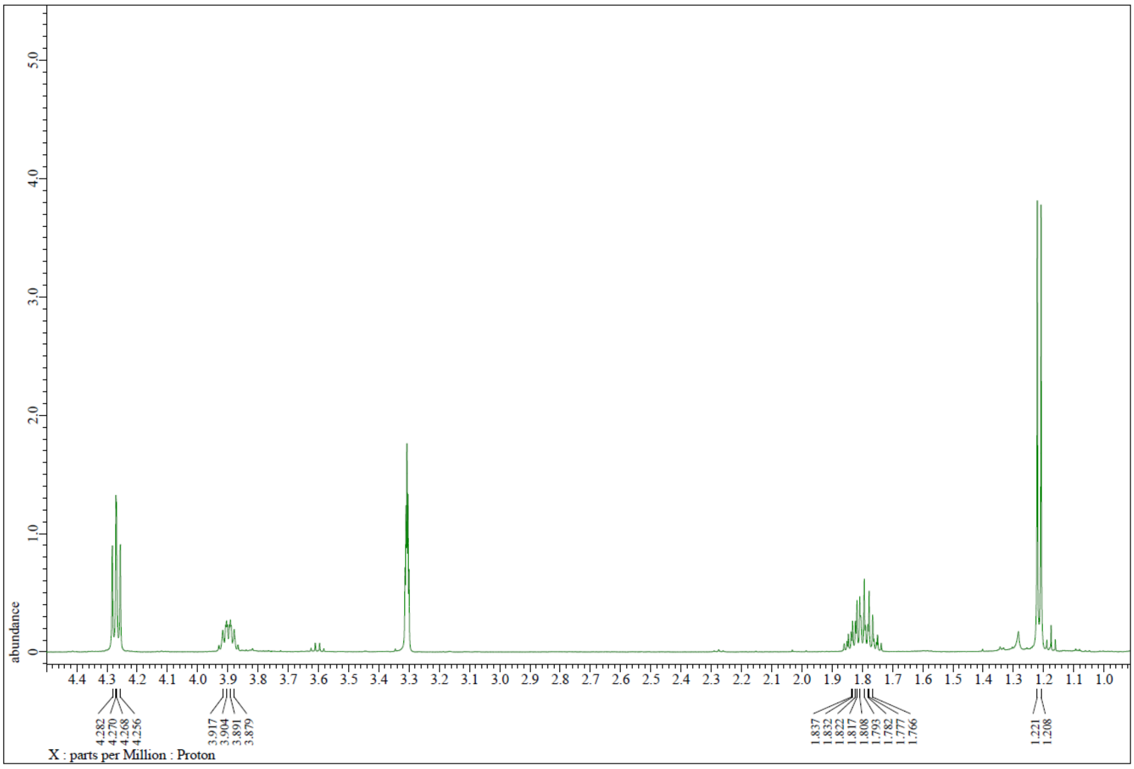

Figure S4. 1H NMR spectrum of Kawarayomogin I at 0.9-4.5 ppm

Figure S5.

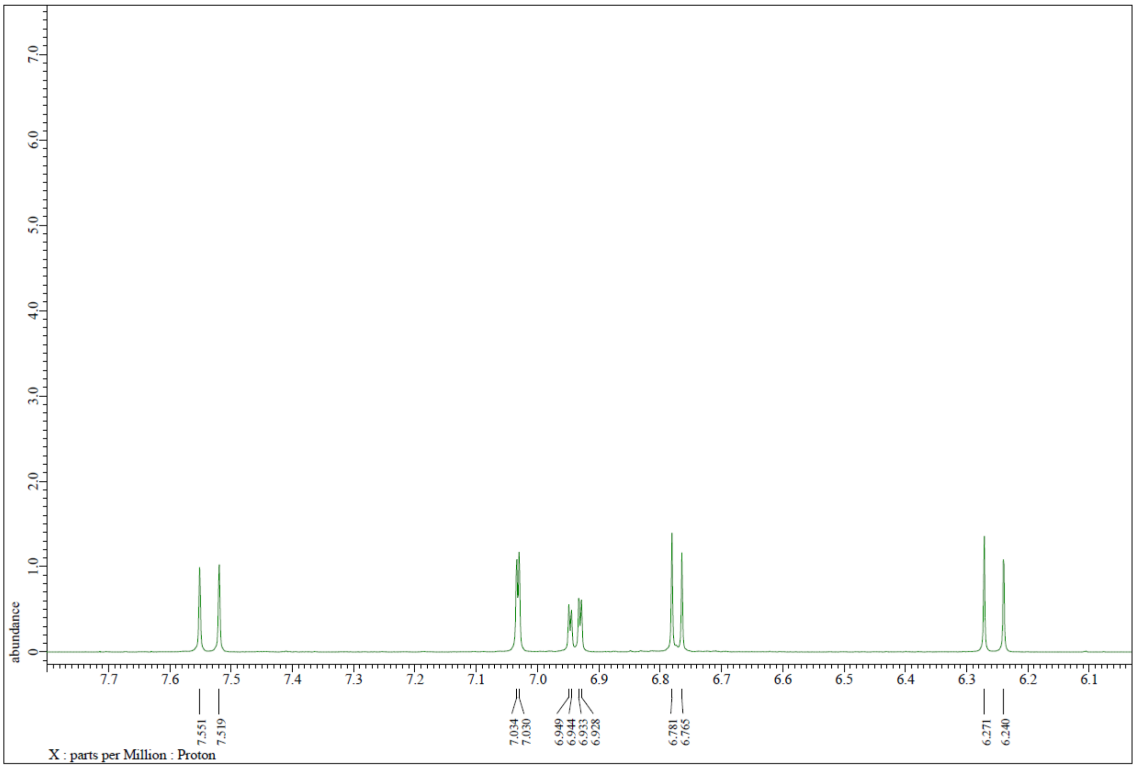

Figure S5. 1H NMR spectrum of Kawarayomogin I at 6.0-7.8ppm

Figure S6.

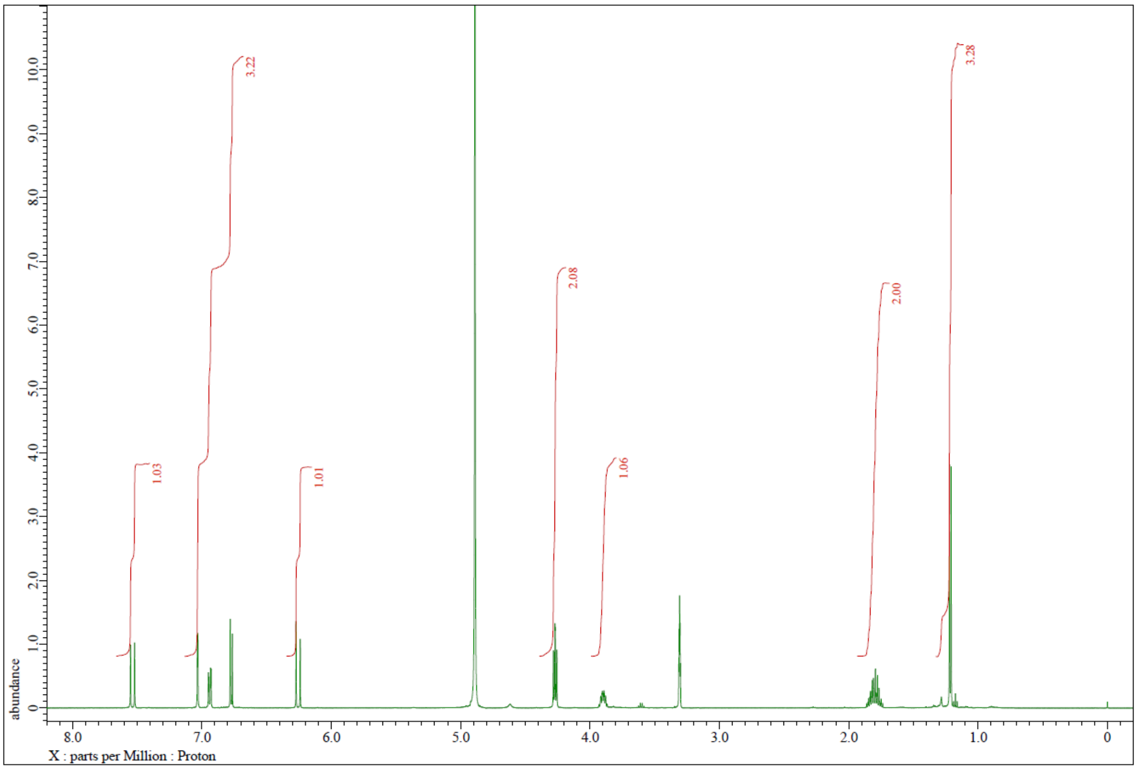

Figure S6. 1H NMR spectrum of Kawarayomogin I with integral curve

Figure S7.

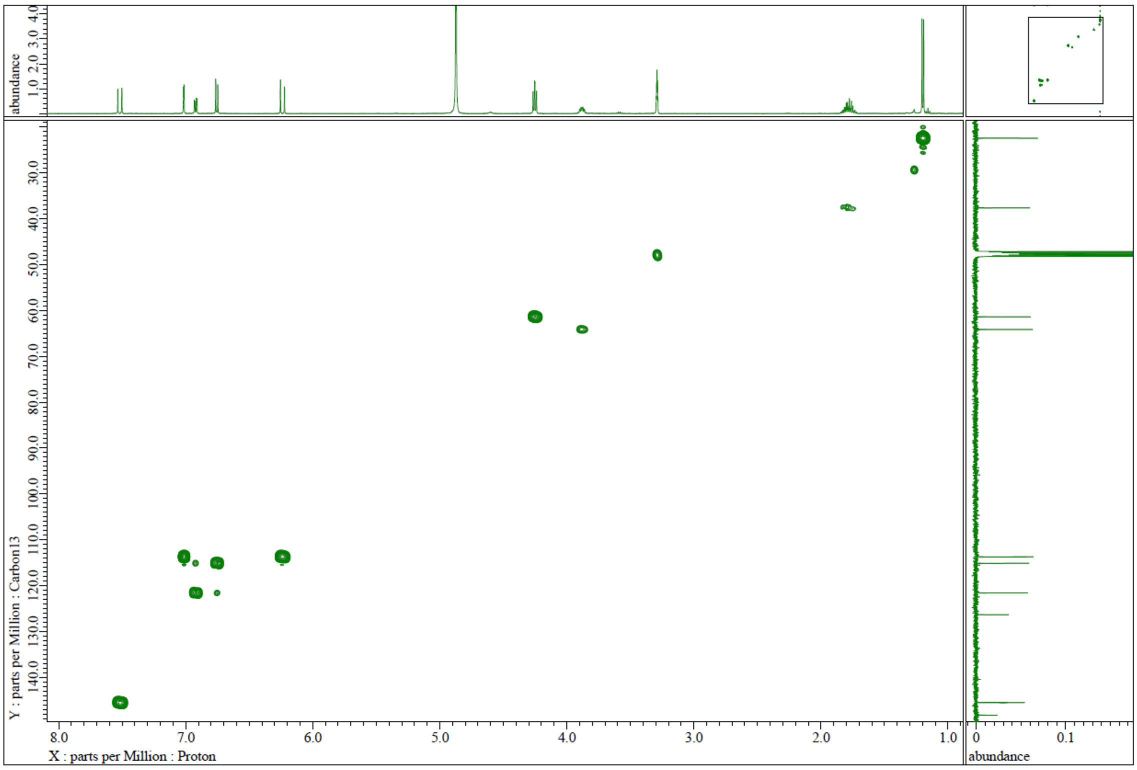

Figure S7. Two-dimensional NMR spectrum (HMQC) data of Kawarayomogin I

Figure S8.

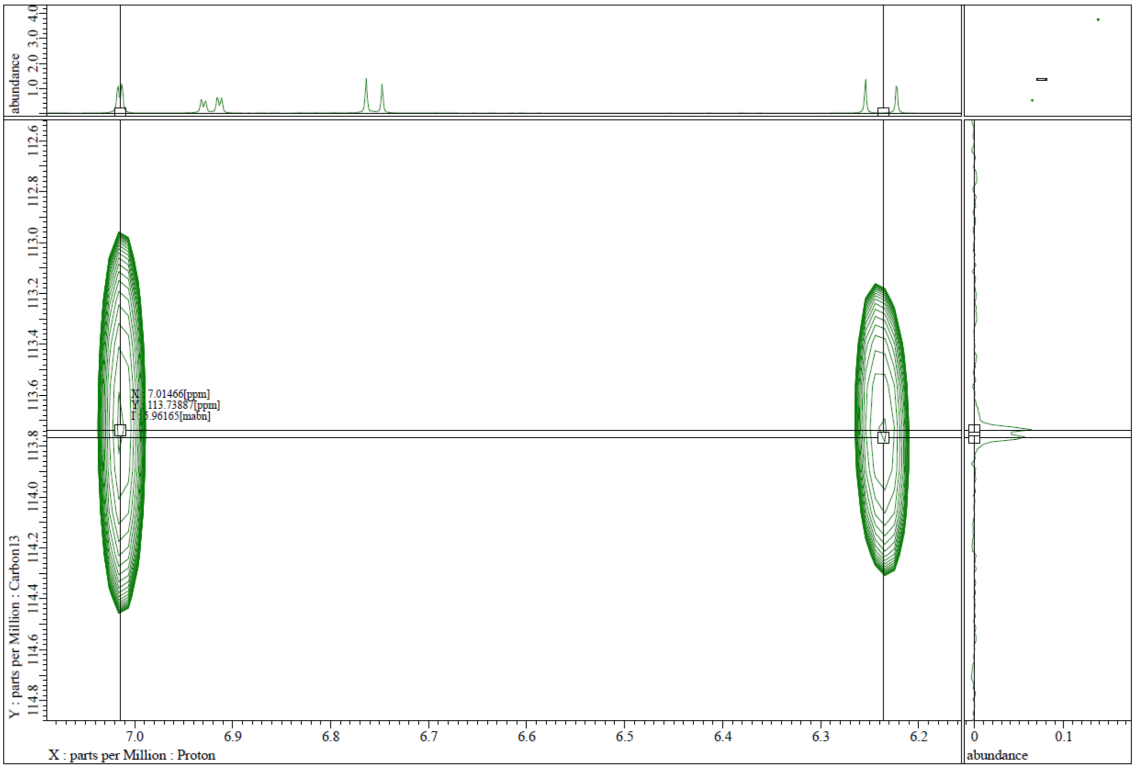

Figure S8. Expanded data of 2D NMR spectrum (HMQC) of Kawarayomogin I at 6.1-7.1ppm for 1H NMR and 112.6-114.8ppm for 13C NMR

Figure S9.

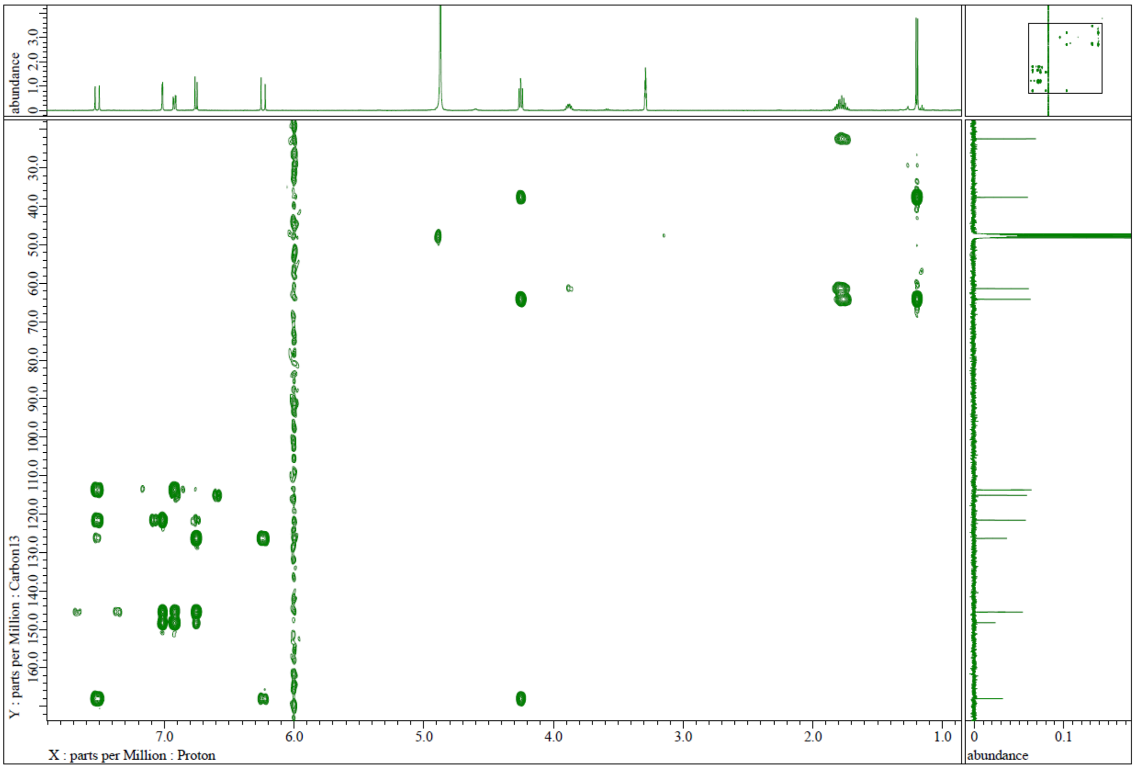

Figure S9. Two-dimensional NMR spectrum (HMBC) data of Kawarayomogin I

Figure S10.

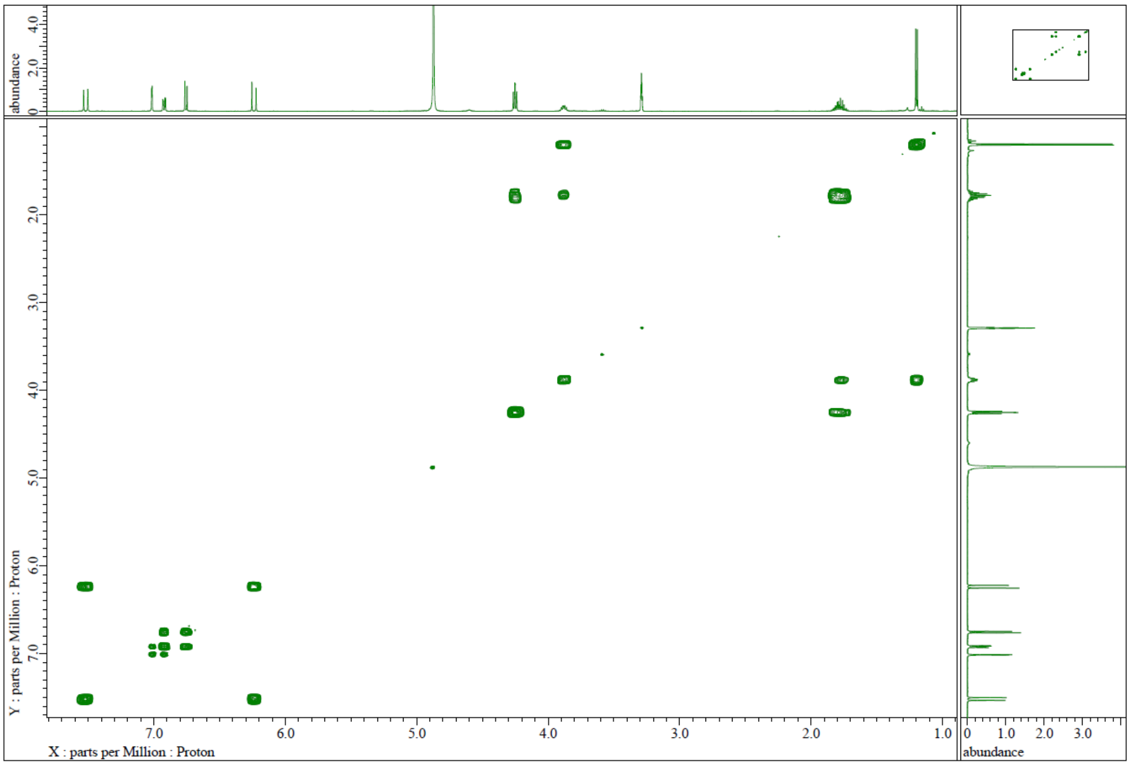

Figure S10. Two-dimensional NMR spectrum (COSY) data of Kawarayomogin I

Figure S11.

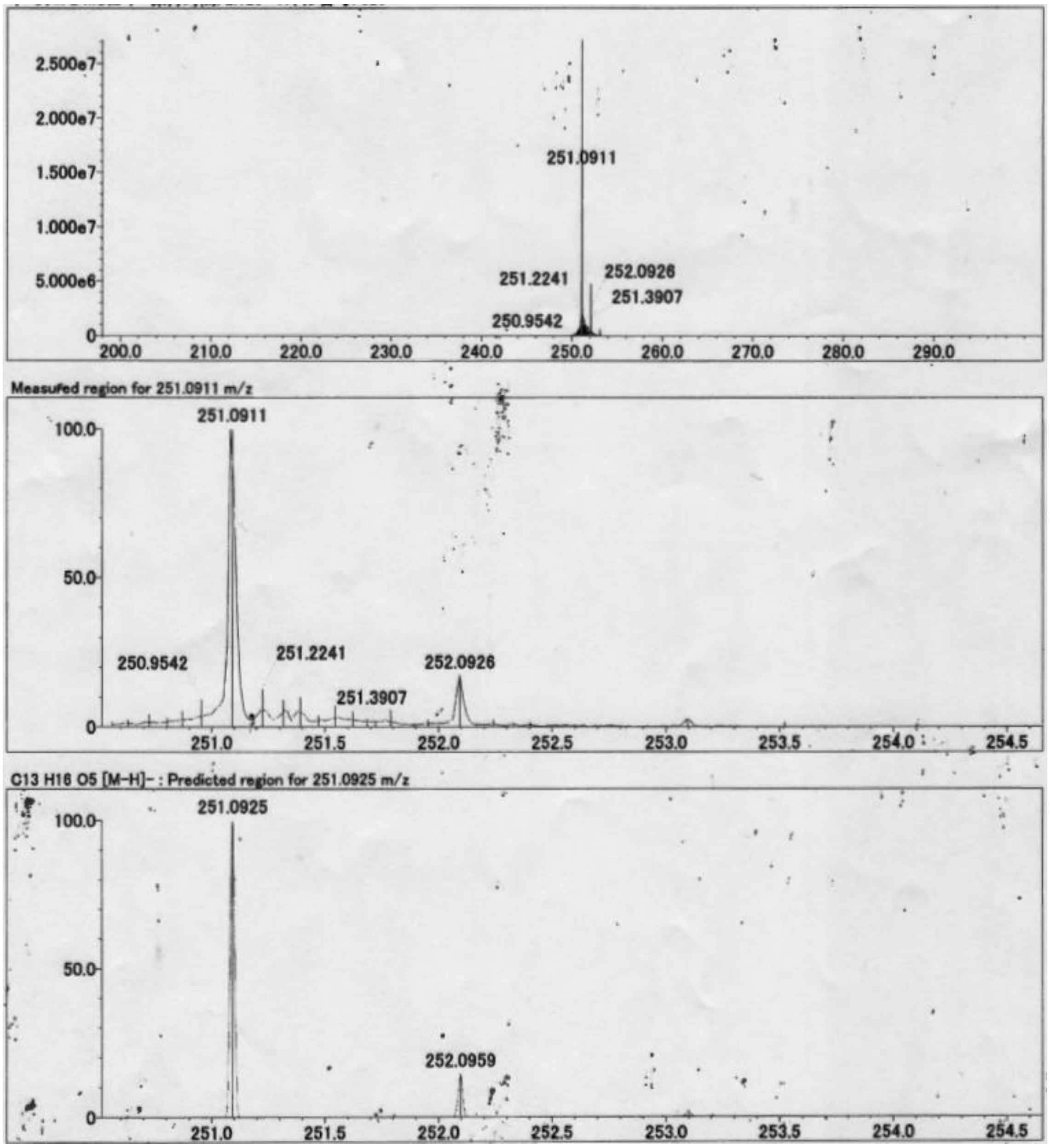

Figure S11. MS spectrum of Kawarayomogin I

Figure S12.

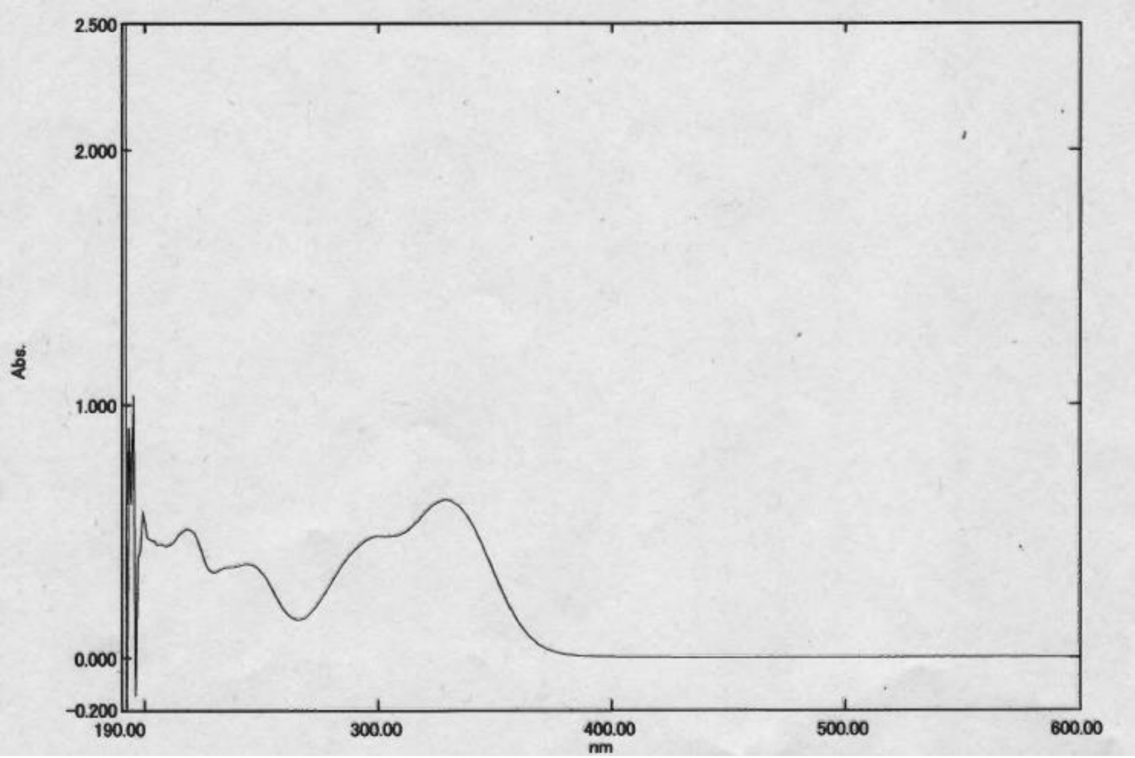

Figure S12. Specific rotational radius of Kawarayomogin I

Figure S13.

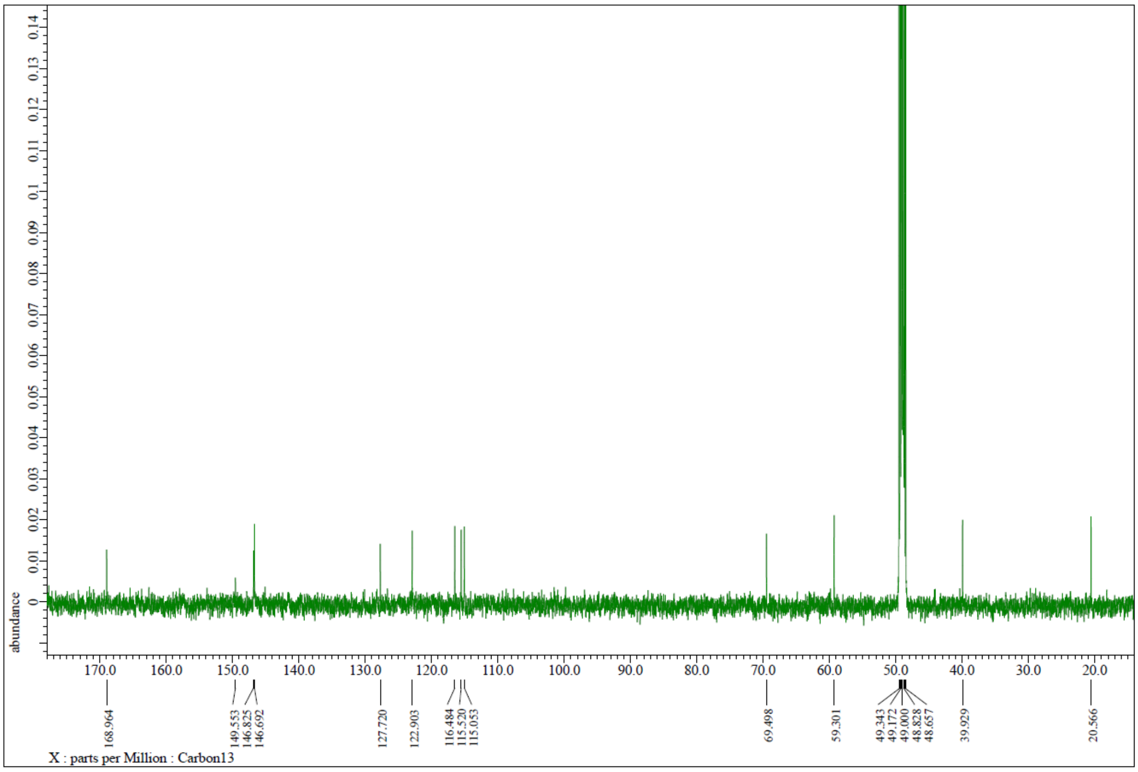

Figure S13. 13C NMR spectrum of Kawarayomogin II

Figure S14.

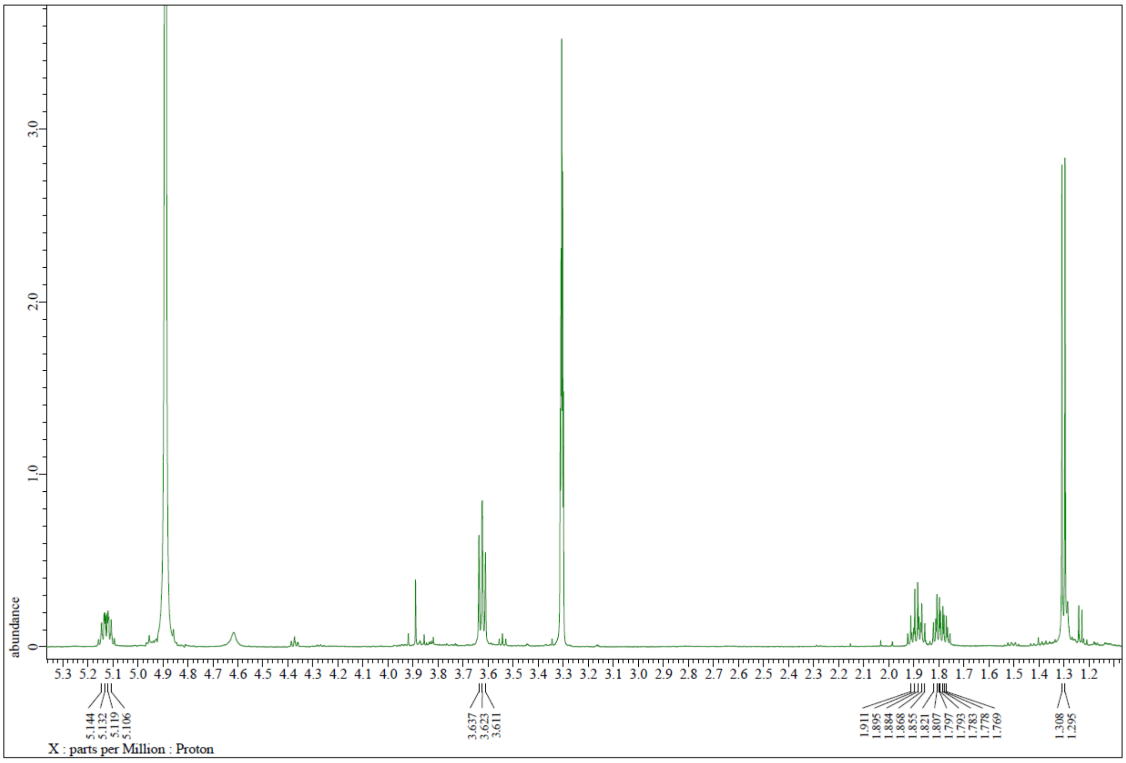

Figure S14. 1H NMR spectrum of Kawarayomogin II at 1.1-5.4 ppm

Figure S15.

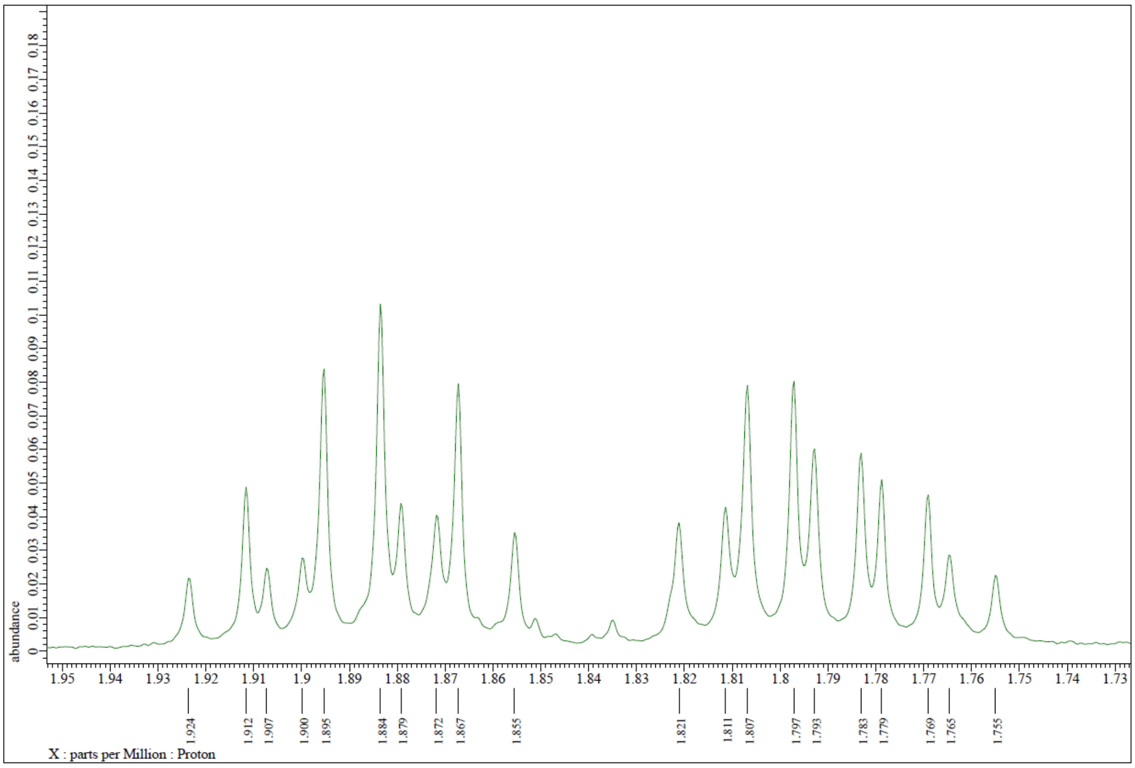

Figure S15. Expanded 1H NMR spectrum of Kawarayomogin II at 1.73-1.95 ppm

Figure S16.

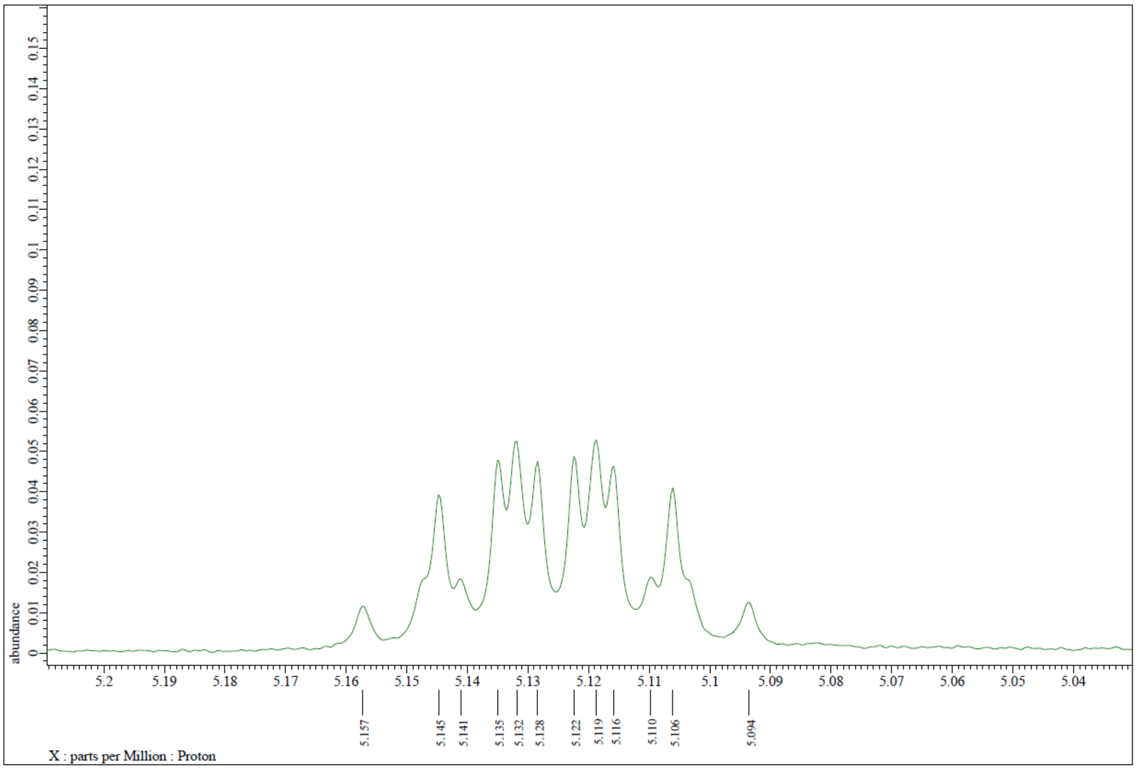

Figure S16. Expanded 1H NMR spectrum of Kawarayomogin II at 5.03-5.21ppm

Figure S17.

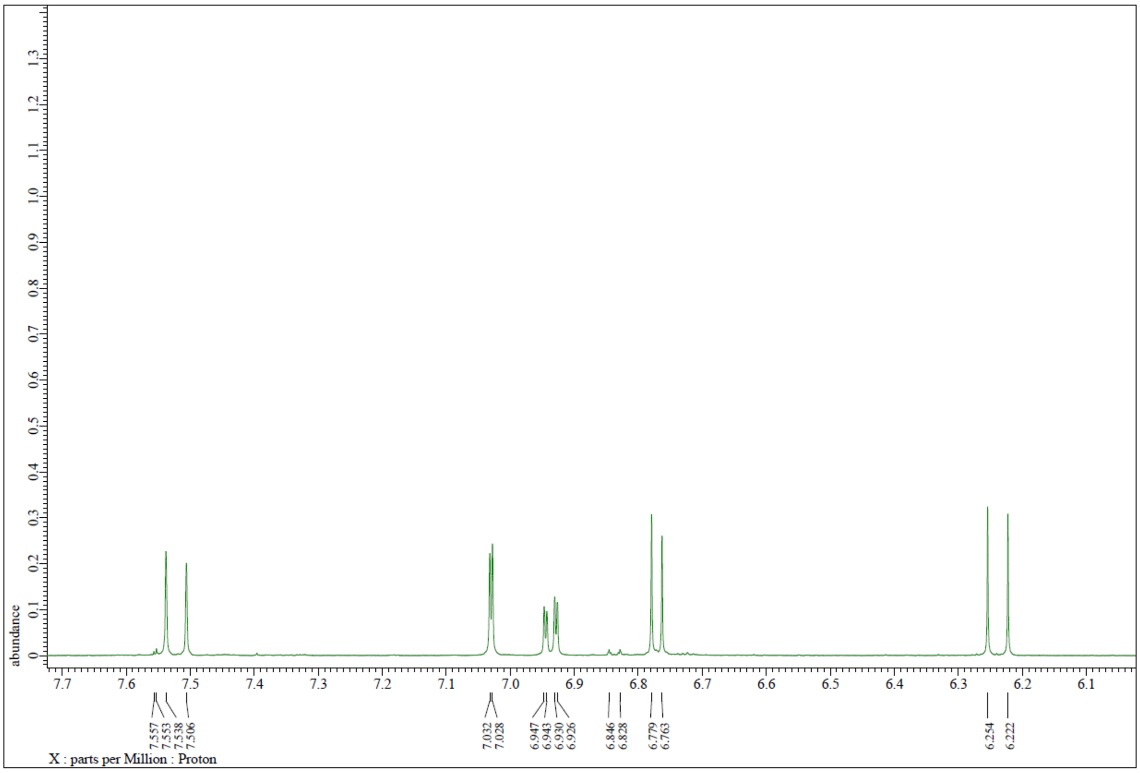

Figure S17. 1H NMR spectrum of Kawarayomogin II at 6.0-7.7 ppm

Figure S18.

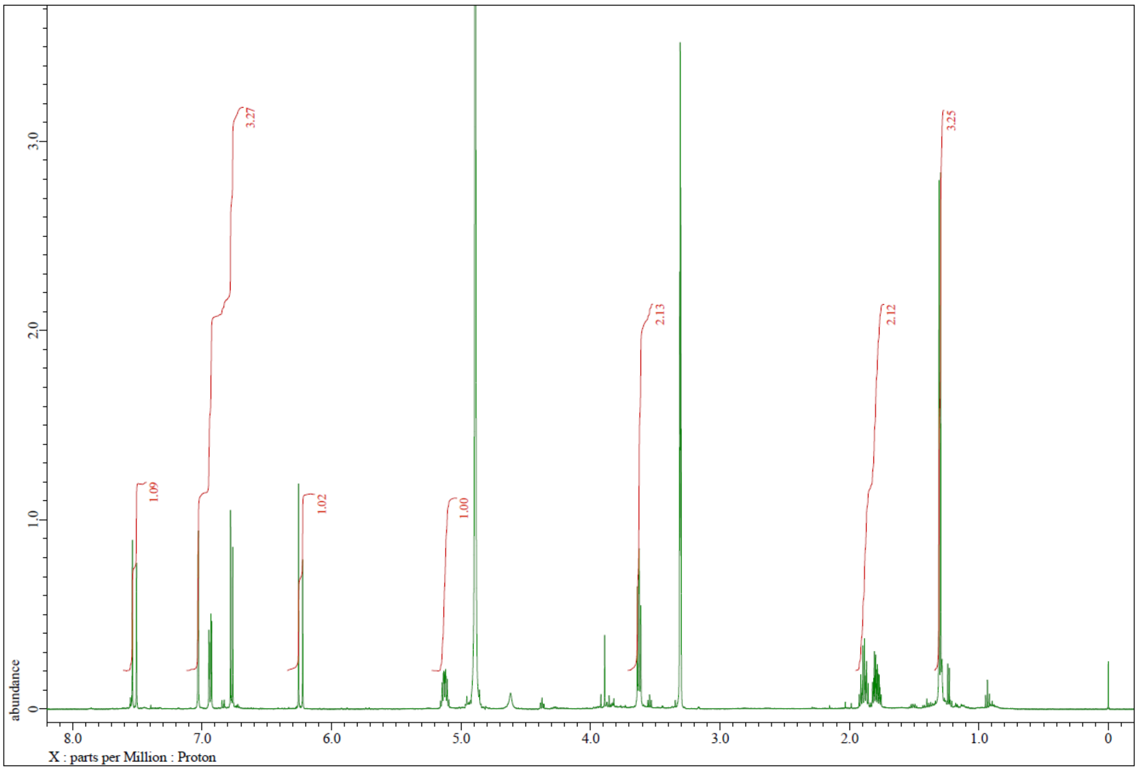

Figure S18. 1H NMR spectrum of Kawarayomogin II with integral curve

Figure S19.

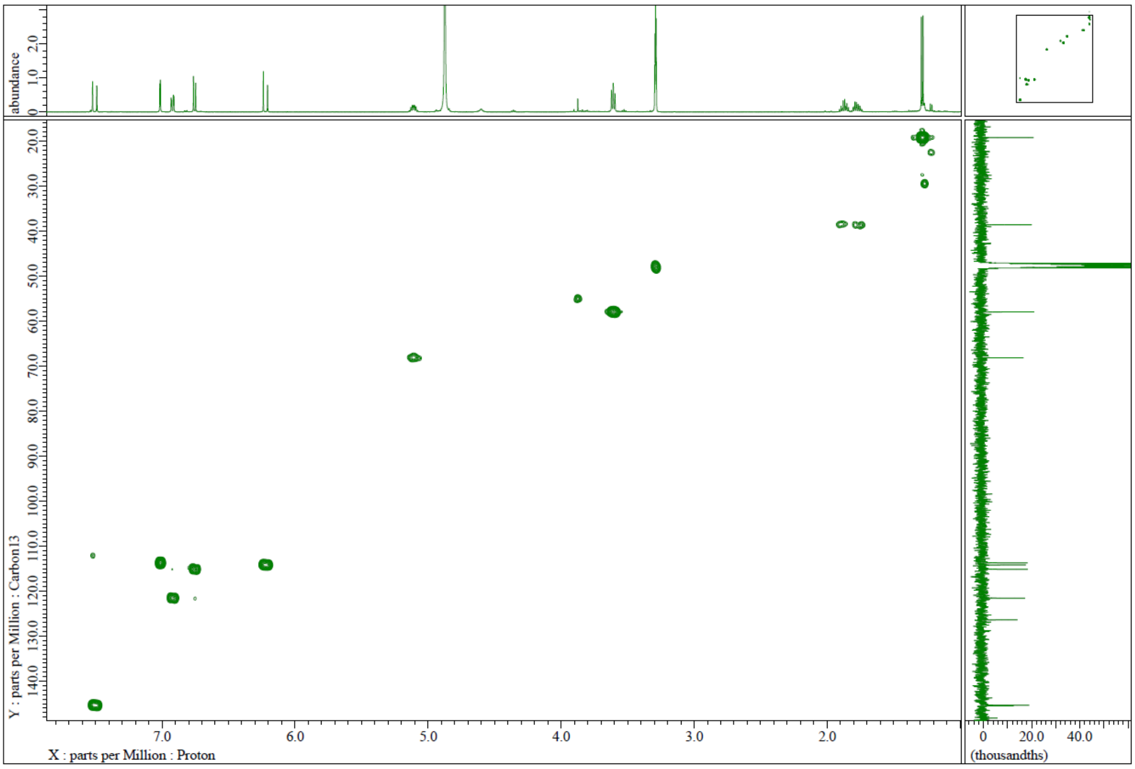

Figure S19. Two-dimensional NMR spectrum (HMQC) data of Kawarayomogin II

Figure S20.

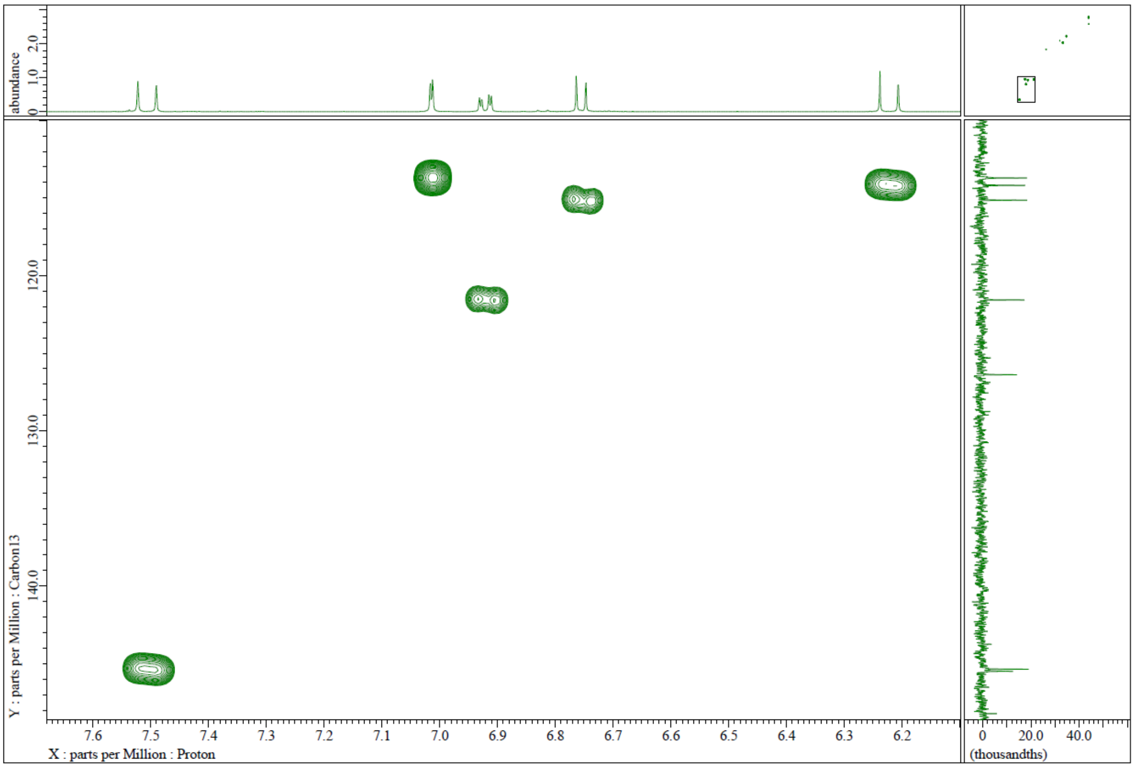

Figure S20. Expanded data of 2D NMR spectrum (HMQC) of Kawarayomogin II at 6.1-7.6 ppm for 1H NMR and 110-150 ppm for 13C NMR

Figure S21.

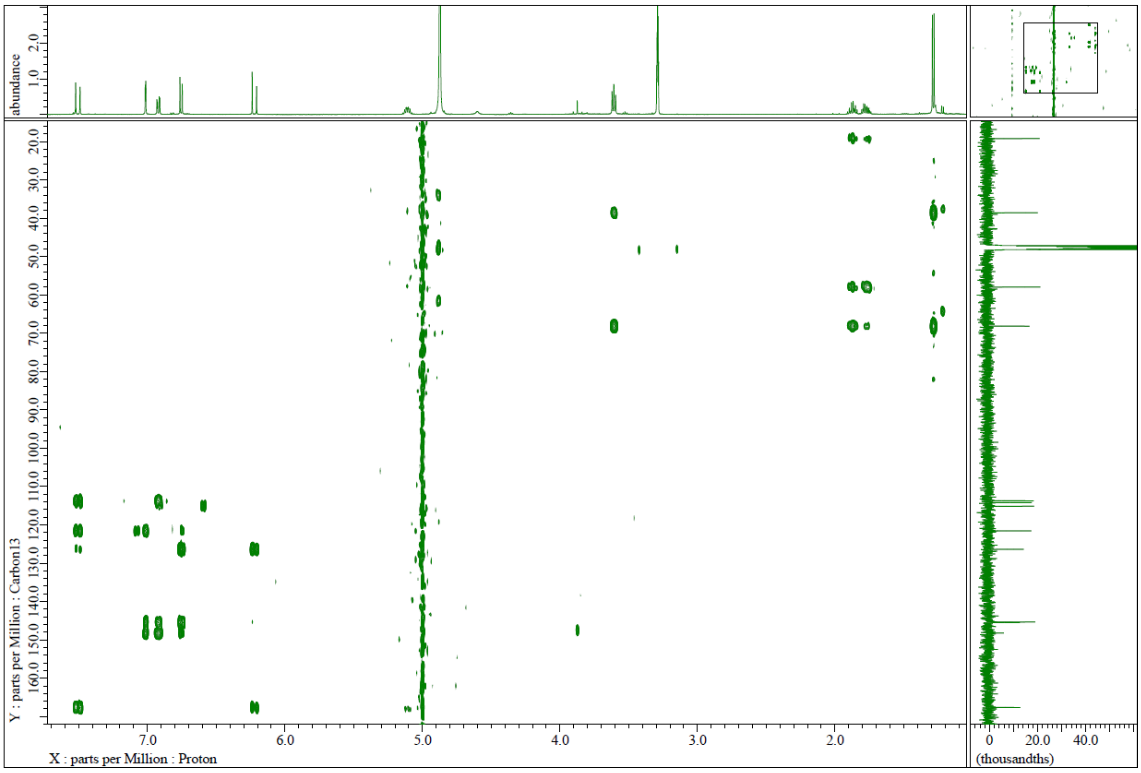

Figure S21. Two-dimensional NMR spectrum (HMBC) data of Kawarayomogin II

Figure S22.

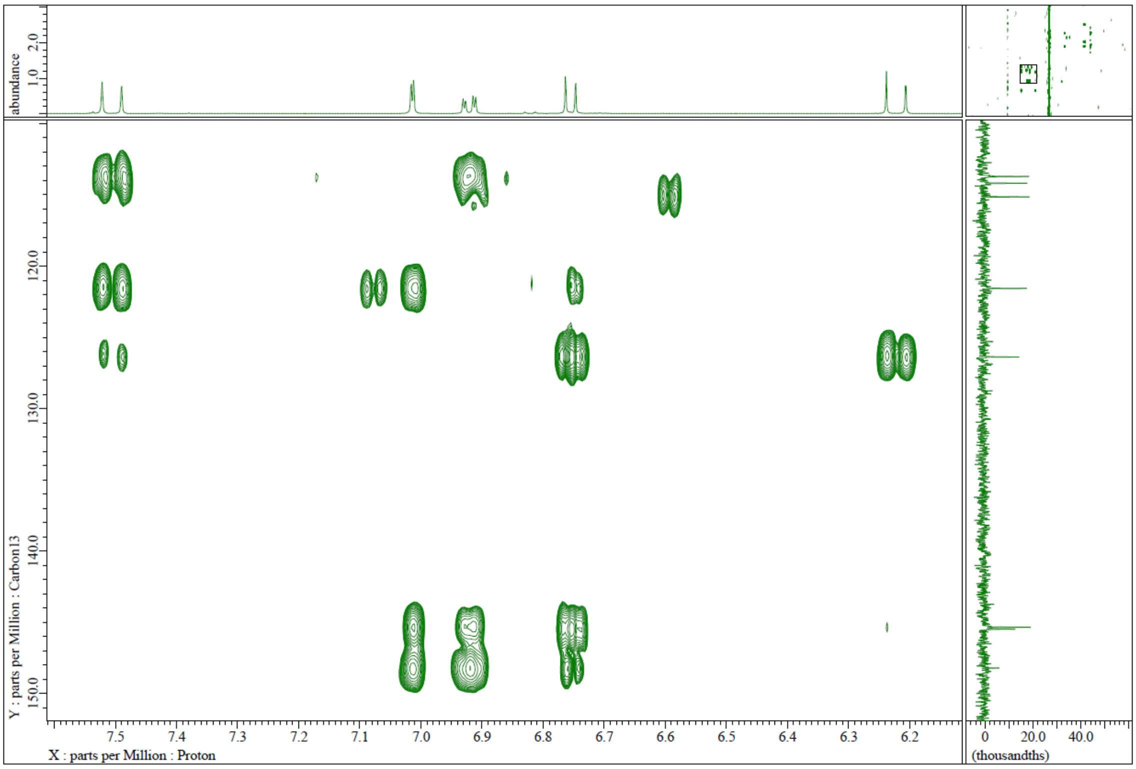

Figure S22. Expanded data of 2D NMR spectrum (HMBC) of Kawarayomogin II at 6.1-7.6 ppm for 1H NMR and 110-150 ppm for 13C NMR

Figure S23.

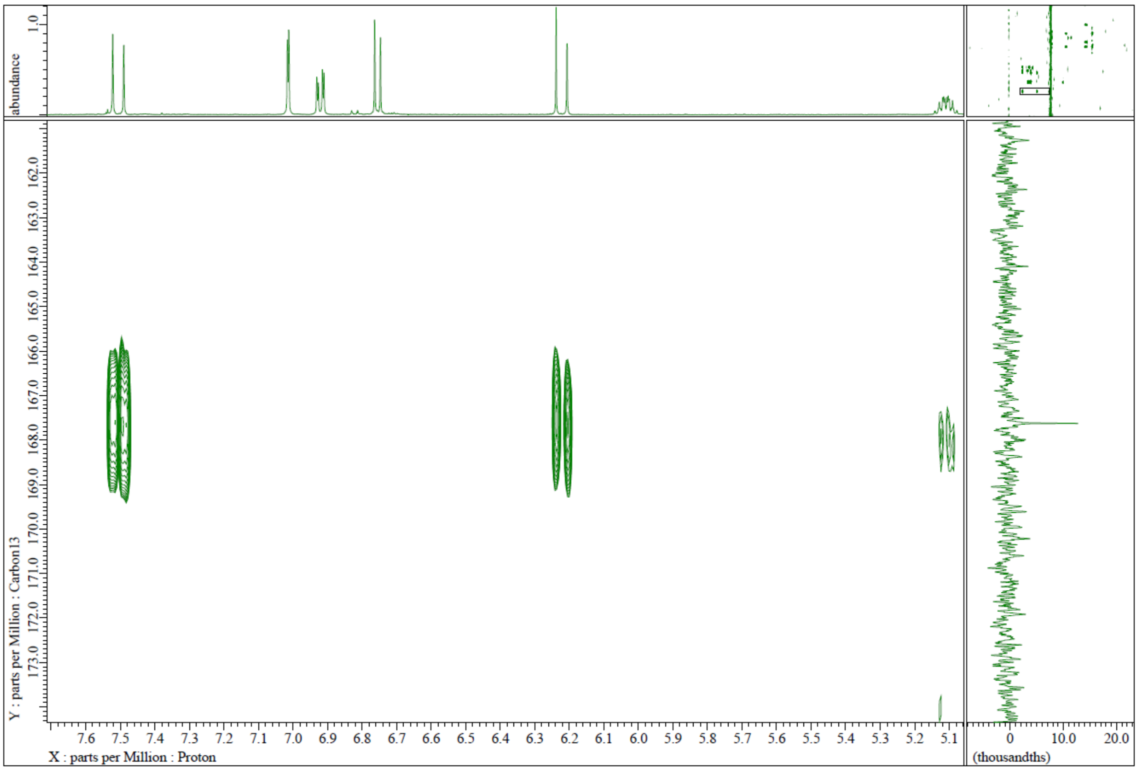

Figure S23. Expanded 2D NMR spectrum (HMBC) of Kawarayomogin II at 5.0-7.7 ppm for 1H NMR and 161-174 ppm for 13C NMR

Figure S24.

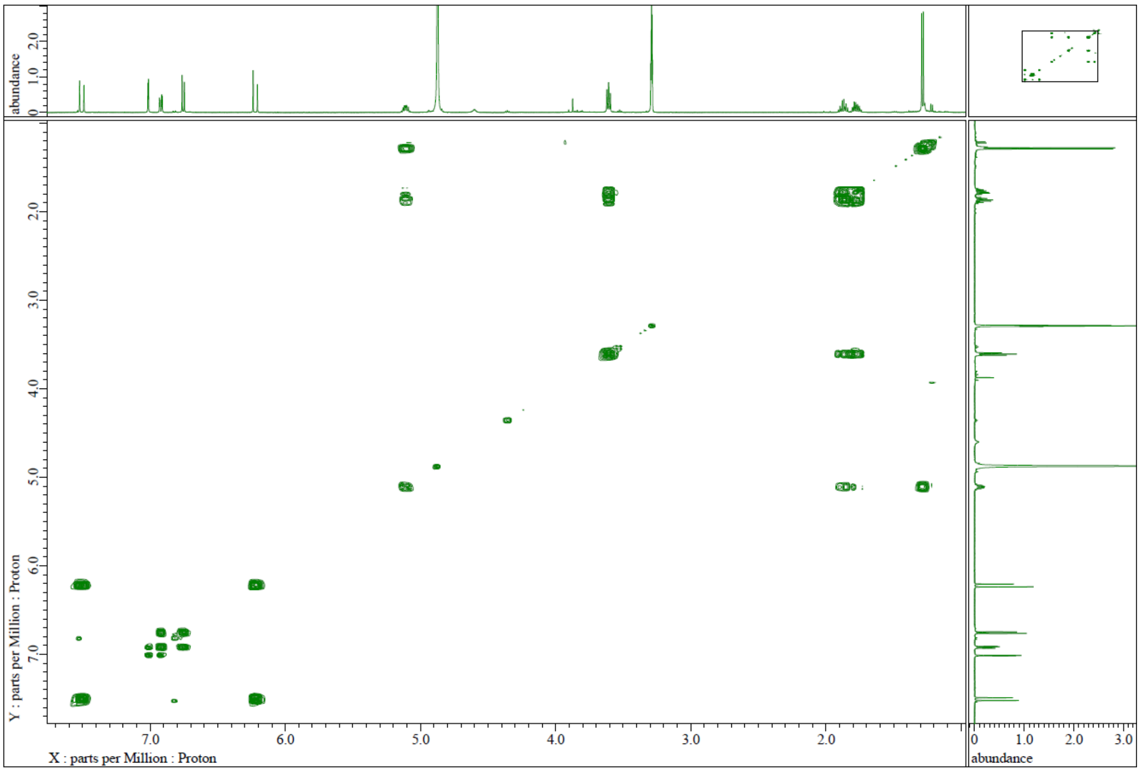

Figure S24. Two-dimensional NMR spectrum (COSY) data of Kawarayomogin II

Figure S25.

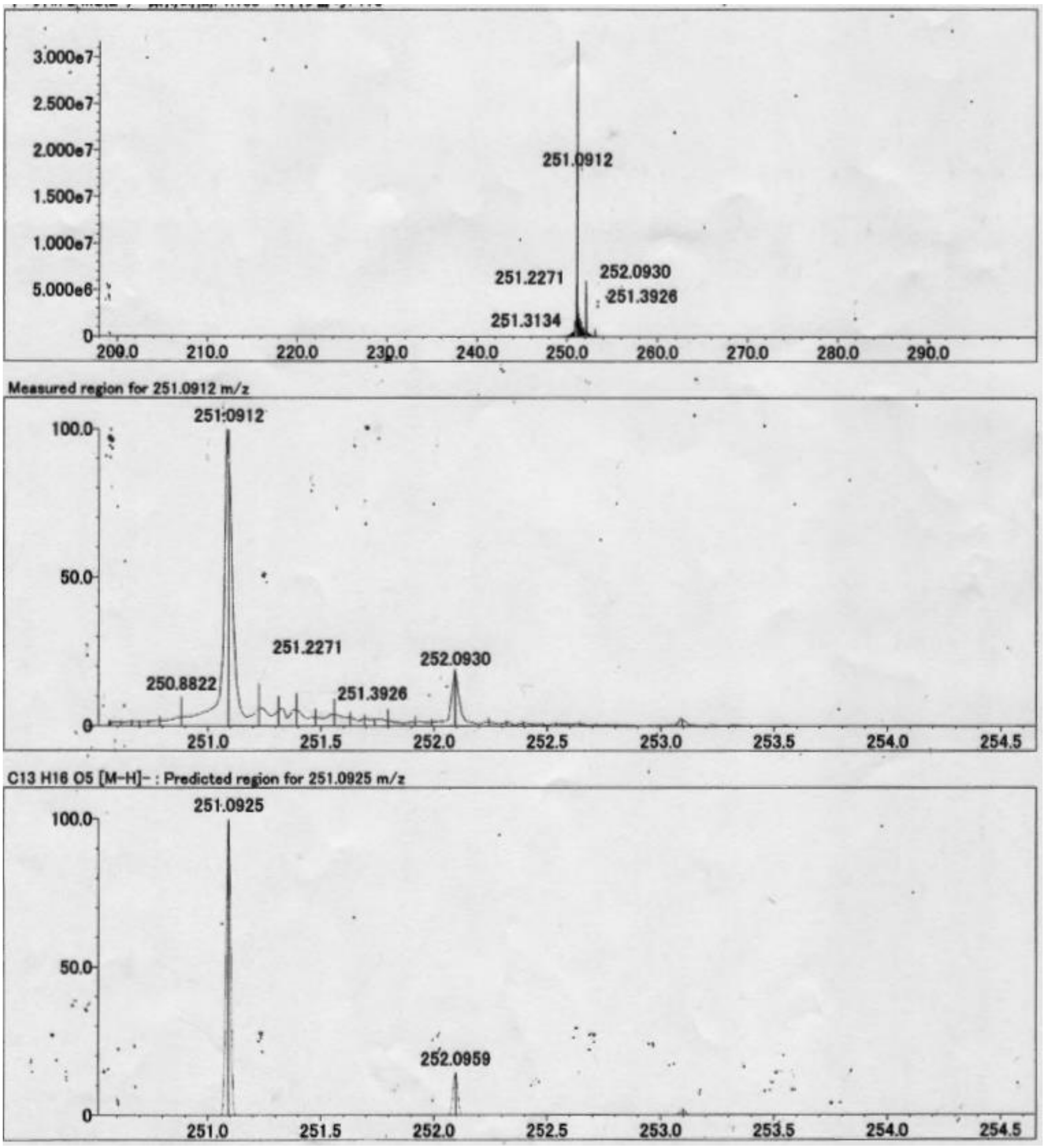

Figure S25. MS spectrum of Kawarayomogin II

Figure S26.

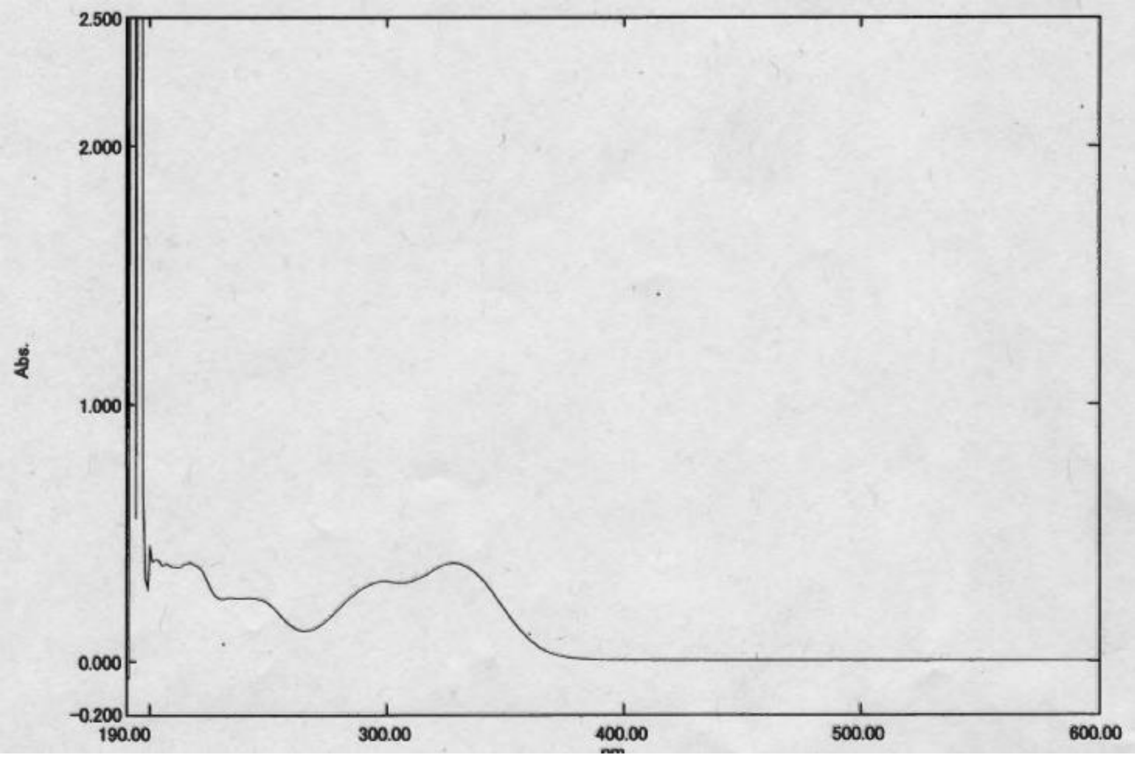

Figure S26. Specific rotational radius of Kawarayomogin II
